# Individual and Combined Association Between Prenatal Polysubstance Exposure and Childhood Risk of Attention-Deficit/Hyperactivity Disorder

**DOI:** 10.1001/jamanetworkopen.2022.1957

**Published:** 2022-03-11

**Authors:** Henri M. Garrison-Desany, Xiumei Hong, Brion S. Maher, Terri H. Beaty, Guoying Wang, Colleen Pearson, Liming Liang, Xiaobin Wang, Christine Ladd-Acosta

**Affiliations:** 1 Department of Epidemiology, Johns Hopkins Bloomberg School of Public Health, Baltimore, Maryland; 2 Center on Early Life Origins of Disease, Department of Population, Family and Reproductive Health, Johns Hopkins University Bloomberg School of Public Health, Baltimore, Maryland; 3 Department of Mental Health, Johns Hopkins University Bloomberg School of Public Health, Baltimore, Maryland; 4 Department of Pediatrics, Boston Medical Center, Boston, Massachusetts; 5 Department of Epidemiology, Harvard T.H. Chan School of Public Health, Boston, Massachusetts; 6 Department of Pediatrics, Johns Hopkins School of Medicine, Baltimore MD; 7 Wendy Klag Center for Autism and Developmental Disabilities, Johns Hopkins University Bloomberg School of Public Health, Baltimore, Maryland

## Abstract

**Importance:**

Polysubstance use among pregnant women has increased because of the opioid epidemic and the increasing legalization of cannabis along with persistent tobacco and alcohol consumption. Previous research on prenatal substance use and the child’s risk of attention-deficit/hyperactivity disorder (ADHD) has mostly focused on single-substance exposures; simultaneous examination of multiple substance use and assessment of their synergistic health consequences is needed.

**Objectives:**

To assess the consequences of the use of specific substances during pregnancy, investigate whether the interaction of multiple prenatal substance exposures is associated with increases in the risk of childhood ADHD, and estimate the aggregate burden of polysubstance exposure during gestation.

**Design, Setting, and Participants:**

This cohort study analyzed data from the Boston Birth Cohort from 1998 to 2019. The sample of the present study comprised a multiethnic urban cohort of mother-child pairs who were predominantly low income. A total of 3138 children who were enrolled shortly after birth at Boston Medical Center were included and followed up from age 6 months to 21 years.

**Exposures:**

Substance use during pregnancy was identified based on self-reported tobacco smoking, alcohol consumption, and use of cannabis, cocaine, or opioids in any trimester of pregnancy. Diagnostic codes for neonatal opioid withdrawal syndrome or neonatal abstinence syndrome from the *International Classification of Diseases, Ninth Revision*, and the *International Classification of Diseases, Tenth Revision*, were also used to identify opioid exposure during gestation.

**Main Outcomes and Measures:**

ADHD diagnosis in the child’s electronic medical record.

**Results:**

Among 3138 children (1583 boys [50.4%]; median age, 12 years [IQR, 9-14 years]; median follow-up, 10 years [IQR, 7-12 years]) in the final analytic sample, 486 (15.5%) had an ADHD diagnosis and 2652 (84.5%) were neurotypical. The median postnatal follow-up duration was 12 years (IQR, 9-14 years). Among mothers, 46 women (1.5%) self-identified as Asian (non–Pacific Islander), 701 (22.3%) as Hispanic, 1838 (58.6%) as non-Hispanic Black, 227 (7.2%) as non-Hispanic White, and 326 (10.4%) as other races and/or ethnicities (including American Indian or Indigenous, Cape Verdean, Pacific Islander, multiracial, other, or unknown). A total of 759 women (24.2%) reported the use of at least 1 substance during pregnancy, with tobacco being the most frequently reported (580 women [18.5%]). Cox proportional hazards models revealed that opioid exposure (60 children) had the highest adjusted hazard ratio (HR) for ADHD (2.19; 95% CI, 1.10-4.37). After including main statistical effects of all individual substances in an elastic net regression model, the HR of opioids was reduced to 1.60, and evidence of a statistical interaction between opioids and both cannabis and alcohol was found, producing 1.42 and 1.15 times higher risk of ADHD, respectively. The interaction between opioids and smoking was also associated with a higher risk of ADHD (HR, 1.17).

**Conclusions and Relevance:**

The findings of this study suggest that it is important to consider prenatal concurrent exposure to multiple substances and their possible interactions when counseling women regarding substance use during pregnancy, the future risk of ADHD for their children, and strategies for cessation and treatment programs.

## Key Points

**Question:**

Is prenatal exposure to multiple substances, alone or in combination, associated with the risk of attention-deficit/hyperactivity disorder (ADHD) in childhood?

**Findings:**

In this cohort study of 3138 children with and without ADHD, prenatal opioid exposure was significantly associated with the highest childhood risk of ADHD among all substances measured. Interactions between exposure to opioids and cannabis as well as opioids and alcohol were also associated with ADHD risk.

**Meaning:**

This study’s findings suggest that it is important to consider prenatal exposure to multiple substances and the interactions between these substances when counseling women regarding substance use during pregnancy.

## Teaser

This cohort study uses data from the Boston Birth Cohort to assess whether prenatal exposure to multiple substances, alone or in combination, is associated with the risk of attention-deficit/hyperactivity disorder in childhood.

## Introduction

Attention-deficit/hyperactivity disorder (ADHD) is a complex neurodevelopmental condition diagnosed in approximately 1 in 10 children as of 2016.^1^ Attention-deficit/hyperactivity disorder has numerous known sequelae that persist from childhood to adulthood, including increased risk of other mental health disorders,^2,3,4,5^ lower employment and educational attainment,^6,7^ and higher all-cause^8,9^ and suicide-specific mortality,^10^ despite recent improvements in treatment strategies. Identification of modifiable risk factors in the prenatal and early-life periods may provide new avenues for prevention efforts. It may also improve early detection of individuals at high risk to complement existing treatment, which could ultimately address disabilities associated with ADHD and improve long-term health outcomes.

Attention-deficit/hyperactivity disorder has known environmental risks in the critical period of development during gestation. Multiple substance exposures during gestation have been associated with the risk of ADHD and other neurological outcomes. Tobacco exposure during pregnancy has been found to have persistent associations with the risk of childhood ADHD,^11^ with 1 study finding an adjusted hazard ratio (HR) of 1.48.^12^ Alcohol consumption^13,14,15^ (1 study^13^ found a relative risk of 1.55) and opioid use (1 study^16^ found that heroin, oxycodone, or opioid agonists used in cessation therapies were associated with an odds ratio of 2.55) have also been associated with increased risk of ADHD. The use of cannabis has been less frequently examined; however, there is some evidence that cannabis is associated with quantitative attention outcomes, although this association has yet to be formally extrapolated to ADHD diagnosis.^17^ Many of these studies, however, had study design limitations, such as recall bias from the use of retrospective designs,^18^ examination of specific populations that may not be generalizable to a wider range of children exposed to substances,^19,20,21^ or lack of adjustment for additional concurrent substance use, which could have produced biased estimates.^17,22^

Polysubstance use is common; an estimated 64.5%^23^ to 89.0%^24^ of pregnant women who use nonmedical opioids also use another substance, and 1 study found that 60% of pregnant women using cannabis had also smoked tobacco in the past month.^25^ Despite these findings, few studies have examined the association of concurrent substance exposure during gestation with ADHD risk among children; most studies have instead grouped polysubstance use with the use of a single substance of interest.^26,27^ In addition, interactions between these substances have not been comprehensively assessed. Only a few studies^13,14^ have assessed the possible interaction between tobacco smoking and alcohol exposure and its association with the risk of ADHD later in childhood.

The objectives of the current study were to assess the consequences of the use of specific substances during pregnancy, investigate whether the possible statistical interaction of multiple prenatal substance exposures was associated with increases in the risk of childhood ADHD, and estimate the aggregate burden of polysubstance exposure during gestation. These data may be used to inform clinicians and public health experts who are engaged in developing intervention strategies to reduce the incidence of ADHD among children. We addressed the limitations of previous studies by including a large multiethnic birth cohort recruited from a safety-net hospital. We used prospective follow-up to assess the risk of ADHD among children exposed to 4 classes of substances (tobacco, alcohol, cannabis, and opioids) to identify possible interactions. Addressing this gap in knowledge may aid in identifying particularly deleterious combinations of substances and provide potential areas of intervention, such as clinical counseling strategies.

## Methods

This cohort study was approved by the institutional review boards of Johns Hopkins School of Public Health and Boston Medical Center. All women provided written informed consent at delivery, and each child provided assent to participate in the study at age-appropriate intervals. A Certificate of Confidentiality (registration No.: CC-HO-11-15) was granted by the National Institutes of Health to ensure participant responses were protected from disclosure to law enforcement agencies. This study followed the Strengthening the Reporting of Observational Studies in Epidemiology (STROBE) reporting guideline for cohort studies.^28^

### Boston Birth Cohort

We conducted a secondary analysis of a prospective cohort using data from the Boston Birth Cohort, which has been described in detail elsewhere.^29^ In brief, the Boston Birth Cohort was initiated in 1998 and is ongoing, comprising approximately 8623 mother-child pairs (or dyads). The present study used data from 1998 to 2019, and the sample was limited to 3156 children with at least 2 years of follow-up (eFigure 1 in the Supplement). Of those, 18 children who were missing data on exposure, outcome, and maternal educational level were excluded because of concerns about reduced precision in multiple imputation given the extent of missingness in their records. The remaining 3138 children were included in the final analytic sample. Multiple-gestation pregnancies, pregnancies resulting from in vitro fertilization, delivery of a newborn with major chromosomal abnormalities, and deliveries resulting from major maternal trauma were excluded. Children (or parents, depending on the child’s age) who continued to receive primary or specialty care at Boston Medical Center were invited to participate beginning at age 6 months until 21 years. A structured questionnaire was administered at baseline by trained research staff; this questionnaire included items about sociodemographic characteristics, substance use, pregnancy history, and health status. Follow-up was conducted through examination of the electronic medical records of children and mothers who continued to receive care at Boston Medical Center.

### Definition of Attention-Deficit/Hyperactivity Disorder

We defined ADHD as a dichotomous case vs control status using physician diagnosis reported in the child’s electronic medical record. Diagnostic codes for ADHD from the *International Classification of Diseases, Ninth Revision* (*ICD-9*; code 314.00 or 314.01) or the *International Classification of Diseases, Tenth Revision* (*ICD-10*; codes F90.0, F90.1, F90.2, F90.8, or F90.9) identified during follow-up were used to define cases. For time-to-event analyses, we used age at first ADHD diagnosis as our outcome variable beginning at age 2 years. Children included in the control group (neurotypical children) did not have any recorded diagnosis of ADHD, autism spectrum disorder, or other developmental disability (*ICD-9* codes 312.xx, 314.02, 314.08, 314.09, or 315.xx and *ICD-10* codes F80.xx-F89.xx). Children in the control group were administratively censored by age (eg, children aged 5 years were censored at 5 years).

### Definition of Substance Use During Pregnancy

Primary substances included maternal use of tobacco products, alcohol, cannabis, and opioids. Information on these substances was collected using a baseline structured questionnaire administered within 24 to 72 hours after delivery. In this questionnaire, women were asked, “In the (first/middle/last) 3 months of your pregnancy, how often did you (smoke/drink/use X drug)?” Women were informed that the study was granted a National Institutes of Health Certificate of Confidentiality, which protected against disclosure of their responses to law enforcement agencies.

We derived a dichotomous prenatal exposed vs unexposed variable for each substance and at each of the following periods: any point in the index pregnancy, trimester 1, trimester 2, or trimester 3. In addition, we used data from the child’s electronic medical record to identify potential neonates exposed to opioids who developed neonatal abstinence syndrome or neonatal opioid withdrawal syndrome (*ICD-9* code 779.5 or *ICD-10* code P96.1). Opioid exposure was identified based on maternal self-report or a diagnosis of neonatal abstinence syndrome or neonatal opioid withdrawal syndrome. Opioid exposure included recreational use of heroin or oxycodone or methadone treatment for opioid use disorder; prescription opioid exposure during pregnancy was not directly measured. Single-substance use was defined as the use of any 1 substance during pregnancy, and polysubstance use was defined as the use of 2 or more substances during pregnancy.

### Polysubstance Exposure

To assess the association of aggregate polysubstance exposure with ADHD risk, we computed a prenatal polysubstance exposure score in 4 ways to better describe estimated synergistic statistical effects between substances. First, using dichotomous yes vs no variables for each exposure, we derived an unweighted additive score equivalent to the number of substances used during pregnancy and in each trimester, ranging from 1 to 4. Second, we derived an alternative exposure score that weighted the summed exposures using bootstrapped estimates from the Cox regression analysis of each substance exposure and the observed ADHD outcome. These bootstrap estimates were generated by splitting the initial data into a training data set (70% of data) for regression estimates and a test data set (30% of data). Third, we validated the pooled estimates in our test data set using bootstrapping for 100 000 iterations across 10 imputed data sets. These independent estimates were pooled and used as the weight for each substance. Fourth, we categorized substance use into 3 groups (none, single substance, or polysubstance) and included these categories in our unadjusted and adjusted Cox proportional hazards models to more broadly understand the global impact of polysubstance use.

### Covariates

We created a directed acyclic graph to select covariates for inclusion in our analysis (eFigure 1 in the Supplement) and measured maternal age, race and ethnicity, marital status, educational level, annual household income, parity, number of perinatal visits, and general stress during pregnancy using a structured interview. Maternal age at delivery was calculated using the mother's birth date. Maternal race and ethnicity information was collected using the following categories: American Indian or Indigenous, Asian (non–Pacific Islander), Cape Verdean, Hispanic, non-Hispanic Black, non-Hispanic White, Pacific Islander, multiracial, or other. Marital status was defined as a categorical variable with 3 groups: never married, currently married, and divorced, widowed, or separated. An ordinal variable for maternal educational level was defined as elementary school, some secondary school, high school graduate, some college, and college graduate. Annual household income was defined as a categorical variable (very low income vs not very low income) using a cutoff of $35 000 per year. Respondents who did not know their annual income were included in a separate *don’t know* category. Parity was coded as a binary variable for nulliparous vs multiparous. The number of prenatal care visits was defined as 0 to 2 visits, 3 to 4 visits, or 5 or more visits. Maternal body mass index (calculated as weight in kilograms divided by height in meters squared) was based on prepregnancy height and weight. Child sex was defined as a binary male vs female variable based on data from the electronic medical record.

### Missingness

Missingness was assessed and hypothesized to be missing at random; therefore, multiple imputation with chained equations was used with 30 iterations to generate 10 data sets. All models were pooled using Rubin rules.^30^

### Statistical Analysis

Descriptive statistics were used to compare children with ADHD with neurotypical children in the control group. Our threshold for significance was 2-sided *P* = .05, and we calculated effect estimates and 95% CIs for all models. All analyses were performed using R software, version 4.0.4 (R Foundation for Statistical Computing).

We used a Cox proportional hazards model and assessed the proportional hazards assumption using Schoenfeld residuals (eFigures 5-14 in the Supplement).^31^ The time measurement used was child age at first ADHD diagnosis. We estimated the association between each individual substance and ADHD diagnosis using separate unadjusted and adjusted models, controlling for major covariates. We then modeled the associations of each substance with ADHD while controlling for additional substance use during pregnancy.

To test for statistical interactions between substances while selecting informative interactions, we used a penalized elastic net regression model.^32^ The α parameter was set at .50 for an elastic net model similar to ridge with least absolute shrinkage and selection operator regression. Using k-fold cross-validation (k = 10), we selected the minimum θ parameter for our models. The θ parameter indicates the amount of shrinkage applied to the coefficients. Each substance was then forced into the models (ie, the penalty was manually set to 0 to ensure that the variable remained in the model). If any higher-order interaction terms (eg, 3-variable interactions) were identified in the model, the lower-order interaction terms (eg, 2-variable interactions) were also forced into the model to assess whether higher-order effects remained. A total of 11 interactions were considered, ranging from 2 variables to all 4 variables. We trained these penalized regression models on 90% of our data and applied the estimated parameters to 10% test data using cross-validation to generate robust estimates of the coefficients. We did not calculate 95% CIs for the HRs derived from the penalized elastic net regression model because penalized estimates artificially reduce variance in estimations by penalizing and shrinking the coefficients, resulting in 95% CIs that are artificially small. Although 95% CIs could have been calculated using bootstrap estimations, we determined it was not appropriate to do so because an impression of high precision may have given when those estimates instead reflected high levels of bias.

As a complementary method to investigate nonlinear interactions between exposures, we assessed the impact of substances while allowing for nonlinear interactions using bayesian kernel machine regression analysis. The probit model was used for binary outcomes using more than 5000 iterations. We generated the posterior inclusion probabilities for each substance and plotted the univariate and bivariate estimator response functions. The overall risks were also estimated, comparing the use of all substances vs no substance during pregnancy based on the model. We plotted univariate and bivariate effects on the H function by substance exposure. The H function is an exposure-response function that allows for nonlinear interactions between exposures to estimate the health outcome; it can be used to calculate single-exposure and interactive risks (eFigures 4 and 5 in the Supplement). It was expected that the results of the bayesian kernel machine regression and penalized regression analyses would reveal patterns for main substance effects that were similar to those of the Cox regression analysis but would also likely reveal a number of interactions that differed from one another because the penalized regression model only used linear interaction terms, whereas the bayesian kernel machine regression analysis included nonlinear exposure effects.

We estimated Pearson correlation coefficients for the substance exposures to assess potential collinearity of exposures (eTable 2 in the Supplement). We also assessed possible sex differences in our Cox regression estimates by stratifying for male vs female (eTable 4 in the Supplement). In addition, we limited our analyses to neurotypical children without any developmental disorder diagnosis as the comparator group vs children with ADHD outcomes (eTable 6 in the Supplement). Given that preterm birth is overrepresented in the Boston Birth Cohort study population, we generated inverse probability weights for preterm birth using major covariates, and we weighted our sensitivity analysis (eTable 7 in the Supplement). We generated E values to estimate the necessary level of confounding that would negate our results (eTable 8 in the Supplement).

## Results

Among 3138 children (1555 girls [49.6%] and 1583 boys [50.4%]; median age, 12 years [IQR 9-14 years]) in the final analytic sample (eTable 1 in the Supplement), 486 (15.5%) had an ADHD diagnosis and 2652 (84.5%) were neurotypical (Table 1). A total of 348 children (71.6%) with ADHD were male compared with 1235 children (42.8%) without ADHD. The median postnatal follow-up duration was 12 years (IQR, 9-14 years). Among mothers, 46 women (1.5%) self-identified as Asian (non–Pacific Islander), 701 (22.3%) as Hispanic, 1838 (58.6%) as non-Hispanic Black, 227 (7.2%) as non-Hispanic White, and 326 (10.4%) as other races and/or ethnicities (including American Indian or Indigenous, Cape Verdean, Pacific Islander, multiracial, other, or unknown). Across the major covariates of interest, mothers of children with ADHD (median age, 28.4 years [IQR, 23.4-33.4 years]) vs mothers of neurotypical children (median age, 26.7 years [IQR, 22.5-33.3 years]) were more likely to be single, divorced, or widowed (365 women [75.1%] vs 1739 women [65.6%]) and less likely to have graduated from college (49 women [10.1%] vs 377 women [14.2%]).

**Table 1.** Characteristics of Participants in the Boston Birth Cohort, 1998-2019

Characteristic	No. (%)	
	No ADHD diagnosis	ADHD diagnosis
Total participants, No.	2652	486
Maternal age, median (IQR), y	26.7 (22.5-33.3)	28.4 (23.4-33.4)
Maternal race and ethnicity		
Asian (non-Pacific Islander)	44 (1.7)	2 (0.4)
Hispanic	589 (22.2)	112 (23.0)
Non-Hispanic Black	1541 (58.1)	297 (61.1)
Non-Hispanic White	194 (7.3)	33 (6.8)
Other^a^	284 (10.7)	42 (8.6)
Maternal marital status		
Single	1671 (63.0)	344 (70.8)
Married	913 (34.4)	121 (24.9)
Other^b^	68 (2.6)	21 (4.3)
Maternal educational level		
No school or elementary school	117 (4.4)	15 (3.1)
Some secondary school	631 (23.8)	127 (26.1)
High school graduate	946 (35.7)	191 (39.3)
Some college	581 (21.9)	104 (21.4)
College graduate	377 (14.2)	49 (10.1)
Annual income, $		
<35 000	1221 (46.0)	236 (48.6)
≥35 000	1165 (43.9)	192 (39.5)
Unknown	266 (10.0)	58 (11.9)
Maternal obesity status		
Normal weight	1130 (42.6)	188 (38.7)
Underweight	112 (4.2)	22 (4.5)
Overweight	680 (25.6)	133 (27.4)
Obese	589 (22.2)	114 (23.5)
Unknown	141 (5.3)	29 (6.0)
BMI, median (IQR)	25.1 (21.8-29.6)	25.7 (22.3-30.0)
Previous births		
0	1516 (57.2)	282 (58.0)
≥1	1136 (42.8)	204 (42.0)
Substance exposure		
None	2041 (77.0)	338 (69.5)
Tobacco	460 (17.3)	120 (24.7)
Alcohol	205 (7.7)	48 (9.9)
Cannabis	100 (3.8)	23 (4.7)
Opioids	49 (1.8)	11 (2.3)
Child sex		
Female	1417 (53.4)	138 (28.4)
Male	1235 (46.6)	348 (71.6)
Child postnatal care visits		
<2	811 (30.6)	128 (26.3)
3-4	891 (33.6)	179 (36.8)
≥5	950 (35.8)	179 (36.8)
Child gestational age at birth		
Term	1908 (71.9)	332 (68.3)
Preterm	744 (28.1)	154 (31.7)
Child follow-up duration, median (IQR), y	12 (9-15)	8 (7-10)

Abbreviations: ADHD, attention-deficit/hyperactivity disorder; BMI, body mass index (calculated as weight in kilograms divided by height in meters squared).

^a^ Includes individuals who self-identified as American Indian or indigenous, Cape Verdean, Pacific Islander, multiracial, or unknown.

^b^ Includes separated, divorced, and widowed.

A total of 759 women (24.2%) reported using at least 1 substance during pregnancy. Tobacco smoking was the most often reported (580 women [18.5%]). Overall, 253 children (8.1%) were exposed to alcohol during pregnancy, 123 children (3.9%) were exposed to cannabis, and 60 children (1.9%) were exposed to opioids. All polysubstance scores had a median of 0; the mean for the unweighted polysubstance score was 0.29 (range, 0-4.00), and the mean for the bootstrap estimate–weighted polysubstance score was 0.26 (range, 0-2.93) (eFigure 2 in the Supplement).

**Figure 1.**
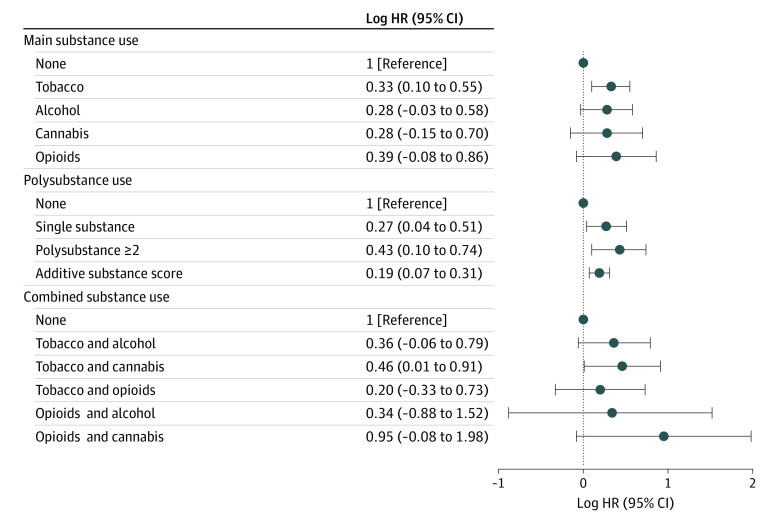
Association Between Prenatal Exposure to Maternal Substance Use and Risk of Childhood Attention-Deficit/Hyperactivity Disorder (ADHD) Hazard ratios (HRs) were derived using a Cox proportional hazards model (log scale is shown). All HRs were adjusted for maternal characteristics (race and ethnicity, age, educational level, marital status, and prepregnancy body mass index [calculated as weight in kilograms divided by height in meters squared]), annual household income quartile, nulliparity, and child sex. The main substance use model (top panel) was adjusted for prenatal exposure to other substances. The polysubstance use model (middle panel) was adjusted for sociodemographic factors.

In the Cox proportional hazards model, opioid exposure had the highest adjusted HR for ADHD (2.19; 95% CI, 1.10-4.37). The log HR for tobacco smoking during pregnancy was 0.33 (95% CI, 0.10-0.55) (Figure 1). After adjusting for other substances, neither cannabis (log HR, 0.28; 95% CI, −0.15 to 0.58) nor alcohol (log HR, 0.28; 95% CI, −0.03 to 0.06) exposure was associated with ADHD. Opioids had the highest effect size in the single-substance adjusted model (log HR, 0.39; 95% CI, −0.08 to 0.86). In the polysubstance models, using a categorical definition yielded a significant increase from a log HR of 0.27 (95% CI, 0.04-0.51) for single-substance exposure to 0.43 (95% CI, 0.10-0.74) for polysubstance exposure. The additive substance score was also significantly associated with ADHD, with an increase in the log HR of 0.19 (95% CI, 0.07-0.31) (eTable 3 in the Supplement). A 21% increase in ADHD risk was found with each additional substance used during pregnancy.

In the penalized elastic net regression analyses, tobacco smoking during pregnancy was included in all models (forced and unforced) (Table 2). After including main statistical effects of all individual substances, the HR of opioids was reduced to 1.60, and the interaction of opioids with both cannabis and alcohol was associated with increased risks of ADHD by HRs of 1.42 and 1.15, respectively. The interaction between opioids and smoking was also associated with a higher risk of ADHD (HR, 1.17). In both the unforced model and the model into which all substance variables were forced, tobacco smoking during pregnancy increased the log HR of ADHD by at least 40% (eTable 5 in the Supplement). Cannabis had the smallest effect size (log HR, 0.14) and was only included in the model when forced. However, opioid use during pregnancy had some statistical interaction with cannabis use, even when forcing all main effects of any substance use into the model. In the fully forced model, opioid use increased the log HR by 23%.

**Table 2.** Penalized Regression Estimates of Main Prenatal Substance Exposure and Exposure Interactions Associated With Childhood Risk of Attention-Deficit/Hyperactivity Disorder

Prenatal exposure	Log HR^a^
Main substance^b^	
Tobacco	0.40
Alcohol	−0.05
Cannabis	0.14
Opioids	0.70
Interaction between substances	
2-Way	
Tobacco and cannabis	Removed
Tobacco and alcohol	Removed
Tobacco and opioids	−0.51
Opioids and cannabis	0.23
Opioids and alcohol	0.17
Alcohol and cannabis	Removed
3-Way	
Tobacco, opioids, and cannabis	0.20
Tobacco, opioids, and alcohol	−0.07
Tobacco, alcohol, and cannabis	Removed
Opioids, alcohol, and cannabis	Removed
4-Way	Removed
Tobacco, opioids, alcohol, and cannabis	Removed

Abbreviation: HR, hazard ratio.

^a^ Hazard ratios from Cox proportional hazards regression analysis using an elastic net model. Some variables were removed from the model during the penalized regression estimation. These variables were not statistically significant, and their estimated effect was essentially null. *P* values were not generated for penalized regression methods because of the bias-variance tradeoff and, given that the model removed uninformative variables, inclusion in the model was considered suggestive of an informative association.^32^ In this analysis, 95% CIs were not calculated because penalized estimates artificially reduce variance in estimations by penalizing and shrinking the coefficients, resulting in 95% CIs that are artificially small and giving the impression of high precision when those estimates instead reflect high levels of bias.

^b^ Main substance effects were accounted for by selecting the option to force them into the model.

**Figure 2.**
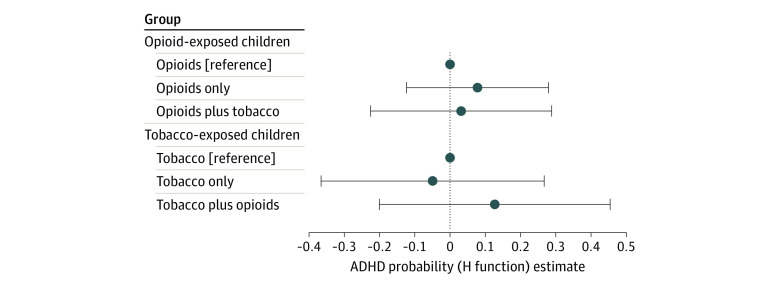
Change in Risk of Attention-Deficit/Hyperactivity Disorder (ADHD) Diagnosis Based on Tobacco and Opioid Exposure Bivariate estimation of change in risk (measured by H function to reflect nonlinear combinations of exposures) for ADHD diagnosis conditional on opioid exposure with and without tobacco exposure and tobacco exposure with and without opioid exposure. Whiskers represent 95% credible intervals.

In the bayesian kernel machine regression probit model, the posterior inclusion probabilities were 0.92 for tobacco smoking, 0.42 for alcohol, 0.42 for opioids, and 0.37 for cannabis. The overall relative risk among those who used all substances increased by 0.78. For the univariate estimated risk using the H function, there was a noticeable increase in risk among those who smoked tobacco (0.08) vs those who did not (0.13) and a slight increase in risk among those who drank alcohol (−0.02) vs those who did not (0.02). Univariate patterns were less clear among those who used cannabis (0.001 vs 0.006) or opioids (−0.007 vs −0.010) vs those who did not (eFigure 3 in the Supplement). In the bivariate models (Figure 2), there was high discrimination between those with and without ADHD, with an estimated change in estimated risk of −0.05 compared with 0.03 in the presence of opioid exposure; a slight upward pattern was observed among those exposed vs not exposed to alcohol (−0.03 vs 0.03) and cannabis (0.0001 vs 0.0020) in the presence of other substances, and a slight downward pattern was observed among those exposed vs not exposed to opioids (0.02 vs −0.01) (eFigure 4 in the Supplement).

## Discussion

To our knowledge, this cohort study is the first to date to examine the association of 4 individual prenatal substance exposures with the risk of ADHD and to systematically assess the consequences of polysubstance exposure. We found ADHD risk was higher among children with prenatal exposure to tobacco smoking or opioids independent of concurrent exposure to other substances. We also found no significant increase in ADHD risk among children with prenatal exposure to cannabis or alcohol after adjusting for exposures to multiple substances. With each additional substance used in pregnancy, there was a 21% increased risk of ADHD, suggesting an additive risk of ADHD with polysubstance exposure. Notably, although we did not find a significant independent association between cannabis exposure and ADHD, children exposed to both cannabis and opioids had a 23% greater risk than expected from either exposure individually. Our results provided information that may be used by clinicians and public health experts to develop more beneficial and efficient intervention strategies to advise pregnant women about the risk of ADHD in their children when these substances are used during pregnancy. This information may be especially important in light of substance use rates in the US; it has been estimated that 5%^33^ of the 3.7 million^34^ estimated children born each year have been exposed to an addictive substance during gestation.

We did not find significant associations between either cannabis or alcohol use and the risk of ADHD when controlling for concurrent substances. With regard to prenatal cannabis exposure, these findings were consistent with recent results from a prospective cohort study^35^ examining all live births in Ontario, Canada, which reported no evidence of an association between cannabis and ADHD. However, cannabis exposure during gestation has been associated with other neurodevelopmental outcomes, including reduced executive and motor function among infants.^36,37^ In addition, prenatal alcohol exposure has previously been reported to have numerous associations with attention-related conditions^38,39^ and remains the etiological exposure associated with fetal alcohol syndrome, which has major developmental consequences. However, a meta-analysis^40^ also found no evidence of a specific association between alcohol exposure and ADHD. It is possible alcohol and cannabis exposures are most pronounced in early life and may not be specifically associated with clinical ADHD, which is typically diagnosed at age 7 years.^41^

This study is also the first, to our knowledge, to report the ordinal associations between increased substance use and the risk of ADHD. Using aggregate measures of polysubstance use, we found an additive statistical effect between the number of substances used during pregnancy and ADHD risk. Use of both weighting schemas yielded similar effect sizes, which added plausibility to our results. These effect sizes likely reflected the aggregate burden which, in the context of a harm reduction framework, may provide some insight regarding possible benefits associated with reducing the use of any substance during pregnancy. Focusing on the most obviously harmful exposures may be a useful way to reduce the risk of ADHD. Further work is needed to directly investigate this hypothesis and examine whether reduction in the use of any substance among those with polysubstance use could be acceptable compared with abstinence.

We also identified important interactions between substances that appeared to increase the risk of ADHD. Only 1 previous study^42^ directly compared the associations of tobacco vs alcohol with the risk of ADHD, and 1 study^13^ examined the consequences of exposure to environmental tobacco smoke among pregnant women who did not themselves smoke and the combination of exposure to environmental tobacco and alcohol. Studies that have examined illicit polysubstance use have only used aggregate measures and have not identified specific interactions associated with ADHD risk.^22,26,27,43^ Our findings suggest opioids may interact with other substances (including cannabis), which may be particularly deleterious. It is not clear whether this interaction is owing to biological or environmental factors, such as whether individuals with illicit polysubstance use are more likely to use more substances or whether they have other characteristics that may impact child development. Future research is needed to further examine potential biological mechanisms.

### Strengths and Limitations

This study has several strengths. The study was conducted among a multiethnic cohort with low income who were at increased risk for both the exposure and outcome. This sample allowed us to identify associations with substances, particularly cannabis and opioids, that other studies may not have had sufficient statistical power to assess. We also had a range of neurodevelopmental outcome data available, which allowed us to identify neurotypical children for inclusion in the control group and reduce the likelihood of misclassification of cases. This study is the first, to our knowledge, to examine outcomes associated with exposure to a myriad of interactive substances within the same cohort. Using innovative methods for environmental mixtures, we modeled additive, multiplicative, and nonlinear interactions among 4 substances to identify prenatal exposure combinations that could be associated with ADHD risk among children.

This study also has limitations. The study used self-reports from mothers to determine exposure status. Because of social desirability bias in reporting, we may have underestimated the amount of substance use in the cohort. However, we obtained a National Institutes of Health Certificate of Confidentiality, which was explained to all mothers, and this certificate barred reporting of their responses to law enforcement agencies. This factor may have improved the accuracy of self-reporting. Previous modeling using data from the Boston Birth Cohort found that fewer than 16% of women who used substances (who were also included in the present study sample) were likely misclassified because of self-reporting.^44^ Although dosing information was not available, future studies could investigate the association between different concurrent substance doses and ADHD risk with further granularity.

In addition, the use of *ICD-9* and *ICD-10* codes to define the primary outcome may have captured severe forms of the disorder and likely missed the spectrum of attention-deficit disorders that may also be associated with substance exposure. This passive ascertainment of outcomes also meant we were not able to directly follow up participants who switched their care from Boston Medical Center, changed residence, or were unavailable for follow-up for other reasons. These participants were simply censored at the child’s current age. However, our supplementary analysis found that the final sample had sociodemographic characteristics similar to those of the baseline sample, suggesting that those who did continue follow-up were likely similar to those in the analytic sample. In addition, there is some evidence that substance use disorders and ADHD are genetically associated,^45^ and shared genetic risks in the sample may have produced confounding by indication rather than identifying a direct association between substance exposure and ADHD risk. Future studies may directly investigate whether polysubstance use as defined in the present study retains shared genetic underpinnings with ADHD and examine the extent to which these genetic factors may explain estimated associations.

## Conclusions

Using innovative statistical methods, this cohort study found that any increase in the number of substances used during gestation was associated with increases in the risk of developing ADHD in later childhood, and exposures to opioids combined with cannabis and opioids combined with tobacco smoking were of particular concern. These findings have public health implications for addressing polysubstance exposure among children during gestation. Future work is needed to identify potential mechanisms through which substances interact and to integrate this understanding into interventions to inform the clinical counseling of women who use substances during pregnancy.

## Notes

Supplement. **eTable 1.** Comparison of Sociodemographic Characteristics Among Cohort Participants Who Completed Baseline Questionnaire vs Those With Electronic Medical Record Follow-up **eTable 2.** Pearson Correlation Matrix of Substance Exposures **eTable 3.** Unadjusted and Adjusted Cox Proportional Hazards Estimates of Polysubstance Scores With Additional Weighting Schema **eTable 4.** Stratification of Single-Substance Cox Proportional Hazards Models and Substance-Adjusted Models by Sex of Child **eTable 5.** Cross-Validated Penalized Regression Models for Training Data Set of All Individual Substances and Their Interactions, Adjusted for Major Covariates **eTable 6.** Unadjusted and Adjusted Cox Proportional Hazards Models Using Individual Substance and Polysubstance Categories With Multiple Imputation Limited to Neurotypical Children as Comparator **eTable 7.** Unadjusted and Adjusted Cox Proportional Hazards Models Using Individual Substance and Polysubstance Categories With Inverse Probability Weighting for Preterm Birth to Correct for Potential Selection Bias in the Baseline Sample Selection **eTable 8.** E Values of Main Substance Effects in Cox Proportional Hazards Model Reflecting Unmeasured Confounding and Exposure Misclassification Bias **eFigure 1.** Directed Acyclic Graph of Substance Exposures, Attention-Deficit/Hyperactivity Disorder Outcomes, and Measured Covariates **eFigure 2.** Unweighted vs Weighted Polysubstance Score by Attention-Deficit/Hyperactivity Disorder Diagnosis **eFigure 3.** Estimated Change in H Function Due to Univariate Substance Exposure Differences **eFigure 4.** Estimated Change in H Function by the Estimated Bivariate Response Conditional on Concurrent Substance Exposure **eFigure 5.** Schoenfeld Residuals of Multiple Imputation Data (Data Set 1) **eFigure 6.** Schoenfeld Residuals of Multiple Imputation Data (Data Set 2) **eFigure 7.** Schoenfeld Residuals of Multiple Imputation Data (Data Set 3) **eFigure 8.** Schoenfeld Residuals of Multiple Imputation Data (Data Set 4) **eFigure 9.** Schoenfeld Residuals of Multiple Imputation Data (Data Set 5) **eFigure 10.** Schoenfeld Residuals of Multiple Imputation Data (Data Set 6) **eFigure 11.** Schoenfeld Residuals of Multiple Imputation Data (Data Set 7) **eFigure 12.** Schoenfeld Residuals of Multiple Imputation Data (Data Set 8) **eFigure 13.** Schoenfeld Residuals of Multiple Imputation Data (Data Set 9) **eFigure 14.** Schoenfeld Residuals of Multiple Imputation Data (Data Set 10)
