# Supplementary material for: Individual and Combined Association Between Prenatal Polysubstance Exposure and Childhood Risk of Attention-Deficit/Hyperactivity Disorder
Source: JAMA Netw Open. 2022 Mar 11;5(3):e221957. doi: 10.1001/jamanetworkopen.2022.1957 (PMC8917426; doi:10.1001/jamanetworkopen.2022.1957)
Supplement: Supplement. — eTable 1. Comparison of Sociodemographic Characteristics Among Cohort Participants Who Completed Baseline Questionnaire vs Those With Electronic Medical Record Follow-up eTable 2. Pearson Correlation Matrix of Substance Exposures eTable 3. Unadjusted and Adjusted Cox Proportional Hazards Estimates of Polysubstance Scores With Additional Weighting Schema eTable 4. Stratification of Single-Substance Cox Proportional Hazards Models and Substance-Adjusted Models by Sex of Child eTable 5. Cross-Validated Penalized Regression Models for Training Data Set of All Individual Substances and Their Interactions, Adjusted for Major Covariates eTable 6. Unadjusted and Adjusted Cox Proportional Hazards Models Using Individual Substance and Polysubstance Categories With Multiple Imputation Limited to Neurotypical Children as Comparator eTable 7. Unadjusted and Adjusted Cox Proportional Hazards Models Using Individual Substance and Polysubstance Categories With Inverse Probability Weighting for Preterm Birth to Correct for Potential Selection Bias in the Baseline Sample Selection eTable 8. E Values of Main Substance Effects in Cox Proportional Hazards Model Reflecting Unmeasured Confounding and Exposure Misclassification Bias eFigure 1. Directed Acyclic Graph of Substance Exposures, Attention-Deficit/Hyperactivity Disorder Outcomes, and Measured Covariates eFigure 2. Unweighted vs Weighted Polysubstance Score by Attention-Deficit/Hyperactivity Disorder Diagnosis eFigure 3. Estimated Change in H Function Due to Univariate Substance Exposure Differences eFigure 4. Estimated Change in H Function by the Estimated Bivariate Response Conditional on Concurrent Substance Exposure eFigure 5. Schoenfeld Residuals of Multiple Imputation Data (Data Set 1) eFigure 6. Schoenfeld Residuals of Multiple Imputation Data (Data Set 2) eFigure 7. Schoenfeld Residuals of Multiple Imputation Data (Data Set 3) eFigure 8. Schoenfeld Residuals of Multiple Imputation Data (Data Set 4) eFigure 9. Schoenfeld Re [file jamanetwopen-e221957-s001.pdf]

Supplementary Online Content

Garrison-Desany HM, Hong X, Maher BS, et al. Individual and combined association between prenatal polysubstance exposure and childhood risk of attention-deficit/hyperactivity disorder. *JAMA Netw Open*. 2022;5(3):e221957. doi:10.1001/jamanetworkopen.2022.1957

eTable 1. Comparison of Sociodemographic Characteristics Among Cohort Participants Who Completed Baseline Questionnaire vs Those With Electronic Medical Record Follow-up

eTable 2. Pearson Correlation Matrix of Substance Exposures

eTable 3. Unadjusted and Adjusted Cox Proportional Hazards Estimates of Polysubstance Scores With Additional Weighting Schema

eTable 4. Stratification of Single-Substance Cox Proportional Hazards Models and Substance-Adjusted Models by Sex of Child

eTable 5. Cross-Validated Penalized Regression Models for Training Data Set of All Individual Substances and Their Interactions, Adjusted for Major Covariates

eTable 6. Unadjusted and Adjusted Cox Proportional Hazards Models Using Individual Substance and Polysubstance Categories With Multiple Imputation Limited to Neurotypical Children as Comparator

eTable 7. Unadjusted and Adjusted Cox Proportional Hazards Models Using Individual Substance and Polysubstance Categories With Inverse Probability Weighting for Preterm Birth to Correct for Potential Selection Bias in the Baseline Sample Selection

eTable 8. E Values of Main Substance Effects in Cox Proportional Hazards Model Reflecting Unmeasured Confounding and Exposure Misclassification Bias

eFigure 1. Directed Acyclic Graph of Substance Exposures, Attention-Deficit/Hyperactivity Disorder Outcomes, and Measured Covariates

eFigure 2. Unweighted vs Weighted Polysubstance Score by Attention-Deficit/Hyperactivity Disorder Diagnosis

eFigure 3. Estimated Change in H Function Due to Univariate Substance Exposure Differences

eFigure 4. Estimated Change in H Function by the Estimated Bivariate Response Conditional on Concurrent Substance Exposure

eFigure 5. Schoenfeld Residuals of Multiple Imputation Data (Data Set 1)

eFigure 6. Schoenfeld Residuals of Multiple Imputation Data (Data Set 2)

eFigure 7. Schoenfeld Residuals of Multiple Imputation Data (Data Set 3)

eFigure 8. Schoenfeld Residuals of Multiple Imputation Data (Data Set 4)

eFigure 9. Schoenfeld Residuals of Multiple Imputation Data (Data Set 5)

eFigure 10. Schoenfeld Residuals of Multiple Imputation Data (Data Set 6)

eFigure 11. Schoenfeld Residuals of Multiple Imputation Data (Data Set 7)

eFigure 12. Schoenfeld Residuals of Multiple Imputation Data (Data Set 8)

© 2022 Garrison-Desany HM et al. *JAMA Network Open*.

eFigure 13. Schoenfeld Residuals of Multiple Imputation Data (Data Set 9)

eFigure 14. Schoenfeld Residuals of Multiple Imputation Data (Data Set 10)

This supplementary material has been provided by the authors to give readers additional information about their work

eTable 1. Comparison of Sociodemographic Characteristics Among Cohort Participants Who Completed Baseline Questionnaire vs Those With Electronic Medical Record Follow-up

Characteristic	Baseline Sample (N=8509)	Sample with Follow-up (N=3138)
Maternal age, median (IQR), y	27.8 (23.0, 32.9)	28.2 (23.2, 33.4)
Missing	0	0
Maternal race/ethnicity		
Asian (non-Pacific Islander)	180 (2.1%)	46 (1.4%)
Hispanic	2423 (28.5%)	701 (22.3%)
Non-Hispanic Black	4031 (47.4%)	1838 (58.6%)
Non-Hispanic White	1006 (11.8%)	227 (7.3%)
Other[a]	869 (10.2%)	326 (10.4%)
Missing	0	0
Maternal marital status		
Single	5220 (61.3%)	1993 (63.5%)
Married	2871 (33.7%)	1024 (32.6%)
Other[b]	249 (2.9%)	89 (2.8%)
Missing	169 (2.0%)	32 (1.0%)
Maternal education		
No school or elementary	526 (6.2%)	132 (4.2%)
Some secondary	2104 (24.7%)	737 (23.5%)
High school graduate	2793 (32.8%)	1137 (36.2%)
Some college	1640 (19.3%)	684 (21.8%)
College graduate	1286 (15.1%)	426 (13.6%)
Missing	160 (1.9%)	22 (0.7%)
Yearly income		
<$35,000 per year	3763 (44.2%)	1457 (46.4%)
≥$35,000 per year	3853 (45.3%)	1357 (43.2%)
Don’t Know	893 (10.5%)	324 (10.3%)
Maternal obesity status		
Normal weight	3797 (44.6%)	1318 (42.0%)
Underweight	370 (4.3%)	134 (4.3%)
Overweight	2156 (25.3%)	813 (25.9%)
Obese	1607 (18.9%)	703 (22.4%)
Missing	579 (6.8%)	170 (5.4%)
BMI, median (IQR)	24.7 (21.6, 28.9)	25.2 (22.0, 29.8)
Previous births		
None	3663 (43.0%)	1340 (42.7%)
≥1	4846 (57.0%)	1798 (57.3%)
Missing	0	0
Substance Exposure		
Smoking	1635 (19.2%)	577 (18.4%)

© 2022 Garrison-Desany HM et al. *JAMA Network Open*.

Missing	73 (0.8%)	19 (0.6%)
Alcohol	736 (8.6%)	249 (7.9%)
Missing	312 (3.7%)	97 (3.1%)
Cannabis	333 (3.9%)	123 (3.9%)
Missing	5 (0.04%)	0 (0.0%)
Opioids	190 (2.2%)	60 (1.9%)
Missing	7 (0.1%)	0 (0.0%)
Child sex		
Female	4272 (50.2%)	1555 (49.6%)
Male	4327 (49.8%)	1583 (50.4%)
Child postnatal care visits		
less than 2 visits	781 (9.2%)	187 (6.0%)
3 to 4 visits	2169 (25.5%)	752 (24.0%)
5 or more visits	2999 (35.2%)	1070 (34.1%)
Missing	2560 (30.1%)	1129 (36.0%)
Child gestational age at birth		
Term	6190 (72.7%)	2237 (71.2%)
Preterm	2308 (27.1%)	897 (28.6%)
Missing	11 (0.1%)	4 (0.1%)

© 2022 Garrison-Desany HM et al. *JAMA Network Open*.

eTable 2. Pearson Correlation Matrix of Substance Exposures

	Opioids	Cannabis	Alcohol
Smoking	0.25	0.29	0.17
Alcohol	0.01	0.15	
Cannabis	0.04		

© 2022 Garrison-Desany HM et al. *JAMA Network Open*.

eTable 3. Unadjusted and Adjusted Cox Proportional Hazards Estimates of Polysubstance Scores With Additional Weighting Schema

	Unadjusted HR (95% CI)	P-value	Adjusted[1] HR (95% CI)	P-value
Unweighted polysubstance score[4]	1.44 (1.27, 1.63)	<0.001	1.34 (1.17, 1.53)	<0.001
Weighted (adj. estimate)[4]	1.32 (1.21, 1.44)	<0.001	1.25 (1.13, 1.38)	<0.001
Weighted (bootstrap estimate)[5]	1.55 (1.35, 1.79)	<0.001	1.43 (1.22, 1.67)	<0.001

Weighting was conducted using the effect estimates for the adjusted hazards for substances, controlling for concurrent substance use.

[5]Weighting was conducted using Cox proportional hazards estimated bootstrapped over 100,000 iterations for each of the 10 MICE datasets.

© 2022 Garrison-Desany HM et al. *JAMA Network Open*.

eTable 4. Stratification of Single-Substance Cox Proportional Hazards Models and Substance-Adjusted Models by Sex of Child

	Single-substance Cox model[1] HR (95% CI)	P-value	Substance-adjusted Cox model[1] HR (95% CI)	P-value
Female Children Only				
Smoking	2.35 (1.12, 4.90)	0.02	1.78 (0.74, 4.29)	0.20
Alcohol	0.86 (0.28, 2.66)	0.79	0.92 (0.29, 2.86)	0.88
Cannabis	3.33 (1.27, 8.78)	0.01	2.15 (0.67, 6.90)	0.20
Opioids	2.02 (0.45, 9.08)	0.36	1.28 (0.26, 0.63)	0.76
Polysubstance score (unweighted)	1.55 (1.23, 1.95)	<0.001		
Polysubstance score (adj. weighted)	1.40 (1.18, 1.66)	<0.001		
Polysubstance score (bootstrap weighted)	1.71 (1.31, 2.24)	<0.001		
	Single-substance Cox model[1] HR (95% CI)	P-value	Substance-adjusted Cox model[1] HR (95% CI)	P-value
Male Children Only				
Smoking	1.15 (0.68, 1.94)	0.61	1.20 (0.68, 2.12)	0.54
Alcohol	1.05 (0.52, 2.13)	0.89	1.03 (0.50, 2.12)	0.93
Cannabis	0.66 (0.16, 2.76)	0.57	0.58 (0.13, 2.55)	0.48
Opioids	1.21 (0.38, 3.86)	0.74	1.11 (0.33, 3.71)	0.86
Polysubstance score (unweighted)	1.24 (1.05, 1.47)	0.01		
Polysubstance score (adj. weighted)	1.19 (1.05, 1.34)	0.007		
Polysubstance score (bootstrap weighted)	1.31 (1.08, 1.59)	0.006		

[1]Adjusted for race/ethnicity, education level, marital status, pre-pregnancy maternal BMI, income quartile,

© 2022 Garrison-Desany HM et al. *JAMA Network Open*.

eTable 5. Cross-Validated Penalized Regression Models for Training Data Set of All Individual Substances and Their Interactions, Adjusted for Major Covariates

Penalization Type:	LASSO	Elastic Net						
Forcing* of variables:	Forcing all	Unforced	Force Smoking	Force Alcohol	Force Cannabis	Force Opioids	Force Alcohol & Opioids	Forcing all main effects
Smoking	1.59	1.32	1.47	1.32	1.31	1.30	1.29	1.59
Alcohol	1.31	1.13	1.13	1.33	1.15	1.13	1.34	1.31
Cannabis	1.08				1.08			1.08
Opioids	1.60	1.02	1.02			2.03	2.04	1.60
Interactions								
Opioids x Cannabis		1.51	1.57	1.67	1.61	1.25	1.29	
Smoking x Cannabis		1.01		1.06		1.01		
Smoking x Opioids x Cannabis		1.16	1.20	1.25	1.16	1.04	1.04	
Alcohol x Cannabis				0.89				
Smoking x Alcohol x Cannabis								
Alcohol x Opioids		1.77	1.88	1.78	1.91	1.002		
Smoking X Alcohol		1.02			1.02	1.04		
Smoking x Opioids		1.62	1.51	1.77	1.70			
Smoking x Alcohol x Opioids						1.11		
Alcohol x Opioids x Cannabis								
Smoking x Alcohol x Opioids x Cannabis								

[1]"Forcing" indicates that these variables were not penalized in the model and remained in the regression regardless of the shrinkage parameter.

[2]Sociodemographic covariates (race/ethnicity, education level, marital status, pre-pregnancy BMI, income, parity, child sex, maternal age) were also included in the model for adjustment with no forcing. Most often, education, race/ethnicity, marital status, and child's sex were retained in the model after variable selection.

© 2022 Garrison-Desany HM et al. *JAMA Network Open*.

eTable 6. Unadjusted and Adjusted Cox Proportional Hazards Models Using Individual Substance and Polysubstance Categories With Multiple Imputation Limited to Neurotypical Children as Comparator

	Unadjusted HR (95% CI)	P-value	Adjusted[1] HR (95% CI)	P-value
Adjusting for no other substances in the models[2]				
Smoking	1.81 (1.47, 2.22)	<0.001	1.58 (1.26, 1.97)	<0.001
Alcohol	1.45 (1.07, 1.96)	0.02	1.33 (0.98, 1.80)	0.07
Cannabis	1.48 (0.97, 2.26)	0.07	1.31 (0.85, 2.01)	0.22
Opioids	2.70 (1.46, 5.00)	0.001	2.19 (1.10, 4.37)	0.03
Adjusting for all other substances in the model[3]				
Smoking	1.70 (1.35, 2.12)	<0.001	1.51 (1.20, 1.92)	<0.001
Alcohol	1.28 (0.94, 1.74)	0.12	1.23 (0.90, 1.68)	0.19
Cannabis	1.00 (0.64, 1.57)	0.99	1.01 (0.64, 1.58)	0.97
Opioids	1.98 (1.05, 3.71)	<0.001	1.95 (0.97, 3.93)	0.06
Polysubstance category				
No substances	Ref		Ref	
1 substance	1.50 (1.20, 1.88)	<0.001	1.33 (1.05, 1.68)	0.02
2+ substances	2.23 (1.64, 3.03)	<0.001	1.90 (1.38, 2.62)	<0.001
Pairwise Substance Combinations				
Smoking + Alcohol	2.20 (1.44, 3.36)	<0.001	1.84 (1.20, 2.84)	0.006
Smoking + Cannabis	1.97 (1.27, 3.07)	0.003	1.76 (1.12, 2.78)	0.01
Smoking + Opioids	3.34 (1.68, 6.64)	<0.001	2.83 (1.22, 6.55)	0.02
Opioids + Alcohol	4.21 (0.98, 18.03)	0.05	3.01 (0.69, 13.19)	0.14
Opioids + Cannabis	4.86 (0.68, 34.64)	0.11	8.16 (1.12, 59.24)	0.04
Additive polysubstance score[4]	1.44 (1.27, 1.63)	<0.001	1.34 (1.17, 1.53)	<0.001

[1]Adjusted for race/ethnicity, education level, marital status, pre-pregnancy maternal BMI, income quartile, nulliparity, maternal age, and sex of the child.

[2]These four models were independent of one another with no adjustment for other substance use in each.

[3]This represents a single model where all substances are included as covariates in order to adjust for exposure to one another.

[4]Ranges from 0 to 4, with each substance equally contributing a value of 1 point if used during pregnancy. This represents the number of substances used during pregnancy.

© 2022 Garrison-Desany HM et al. *JAMA Network Open*.

eTable 7. Unadjusted and Adjusted Cox Proportional Hazards Models Using Individual Substance and Polysubstance Categories With Inverse Probability Weighting for Preterm Birth to Correct for Potential Selection Bias in the Baseline Sample Selection

	Cox model HR (95% CI)	P-value	Substance-adjusted Cox Model HR (95% CI)	P-value
Unadjusted				
Smoking	1.51 (1.22, 1.85)	<0.001	1.46 (1.16, 1.83)	0.001
Alcohol	1.42 (1.05, 1.91)	0.02	1.27 (0.94, 1.73)	0.12
Cannabis	1.32 (0.86, 2.03)	0.20	0.99 (0.63, 1.56)	0.98
Opioids	1.28 (0.70, 2.34)	0.42	0.99 (0.53, 1.84)	0.98
Polysubstance score (unweighted)	1.28 (1.13, 1.44)	<0.001		
Adjusted				
Smoking	1.34 (1.07, 1.68)	0.010	1.30 (1.02, 1.64)	0.03
Alcohol	1.31 (0.97, 1.77)	0.08	1.22 (0.90, 1.66)	0.20
Cannabis	1.26 (0.82, 1.94)	0.30	1.05 (0.67, 1.65)	0.83
Opioids	1.15 (0.60, 2.20)	0.68	1.02 (0.53, 1.97)	0.95
Polysubstance score (unweighted)	1.21 (1.06, 1.38)	0.005		

© 2022 Garrison-Desany HM et al. *JAMA Network Open*.

eTable 8. E Values of Main Substance Effects in Cox Proportional Hazards Model Reflecting Unmeasured Confounding and Exposure Misclassification Bias

	E-value
Smoking only	1.39
Alcohol only	1.20
Cannabis only	1.01
Opioids only	1.65
Single substance	1.27
Polysubstance (any)	1.62
Additive score (unweighted)	1.28
Smoking and alcohol	1.59
Smoking and cannabis	1.54
Smoking and opioids	2.08
Opioids and alcohol	2.15
Opioids and cannabis	3.76

© 2022 Garrison-Desany HM et al. *JAMA Network Open*.

eFigure 1. Directed Acyclic Graph of Substance Exposures, Attention-Deficit/Hyperactivity Disorder Outcomes, and Measured Covariates

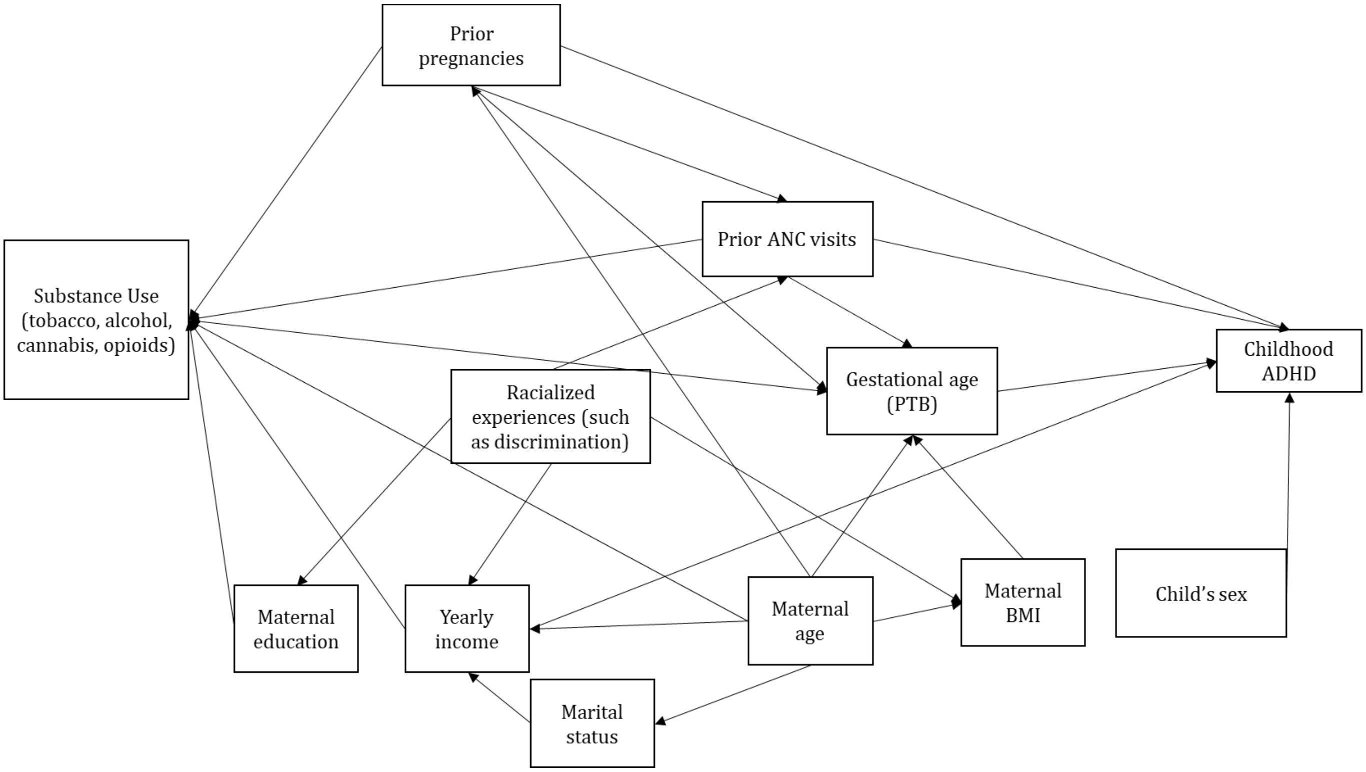

© 2022 Garrison-Desany HM et al. *JAMA Network Open*.

eFigure 2. Unweighted vs Weighted Polysubstance Score by Attention-Deficit/Hyperactivity Disorder Diagnosis

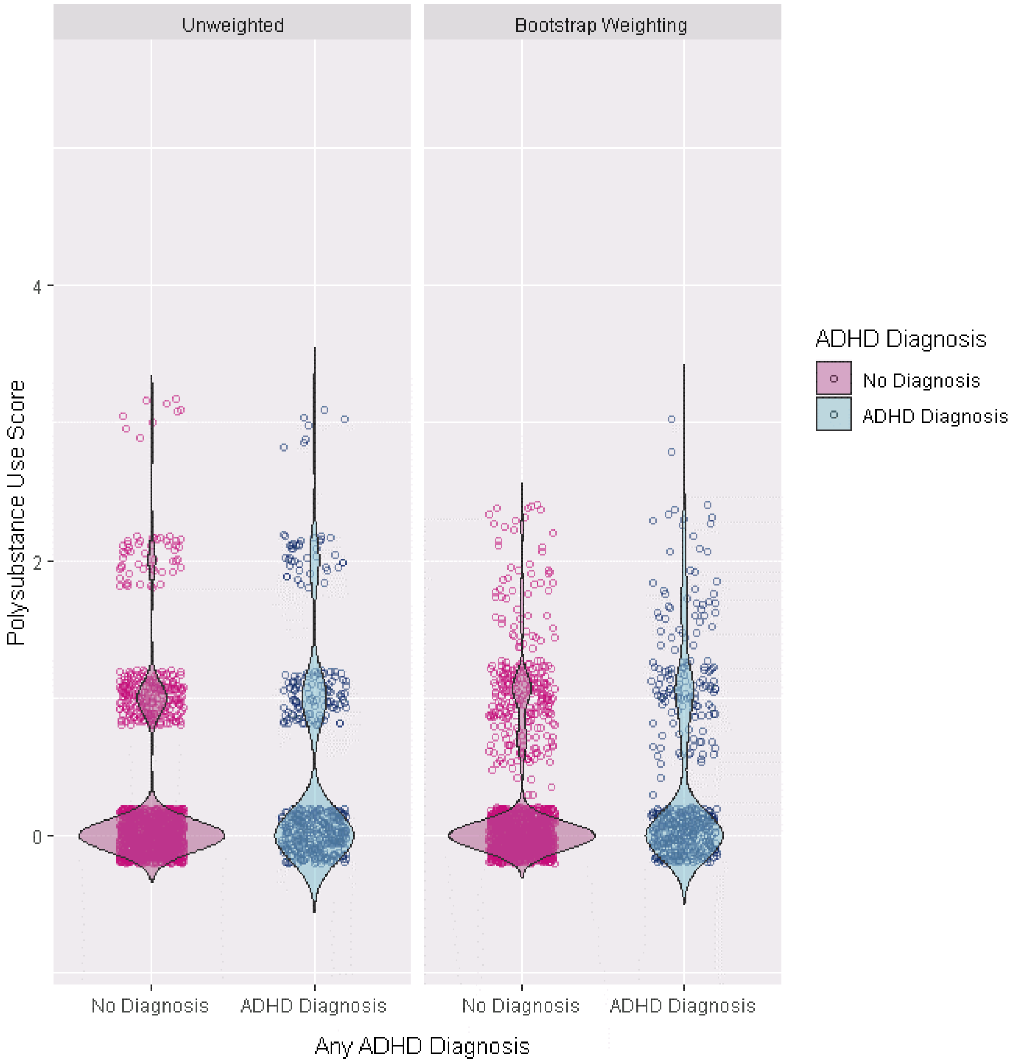

Panels: Unweighted: Unweighted polysubstance score was the sum of substances used during pregnancy. Bootstrap Weighting: Weighting was conducted using Cox proportional hazards estimated bootstrapped over 10,000 iterations for each of the 10 MICE datasets.

© 2022 Garrison-Desany HM et al. *JAMA Network Open*.

eFigure 3. Estimated Change in H Function Due to Univariate Substance Exposure Differences

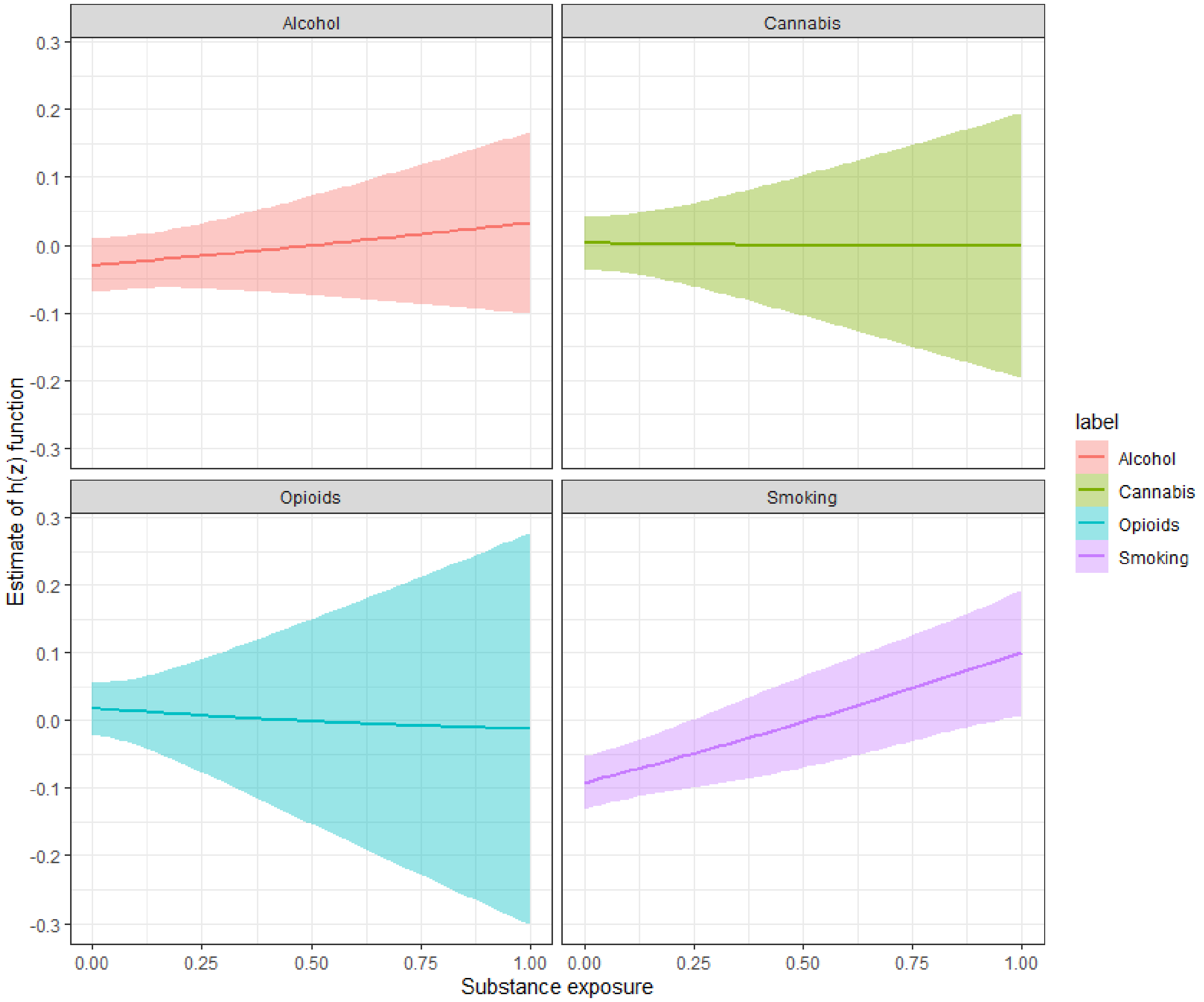

© 2022 Garrison-Desany HM et al. *JAMA Network Open*.

eFigure 4. Estimated Change in H Function by the Estimated Bivariate Response Conditional on Concurrent Substance Exposure

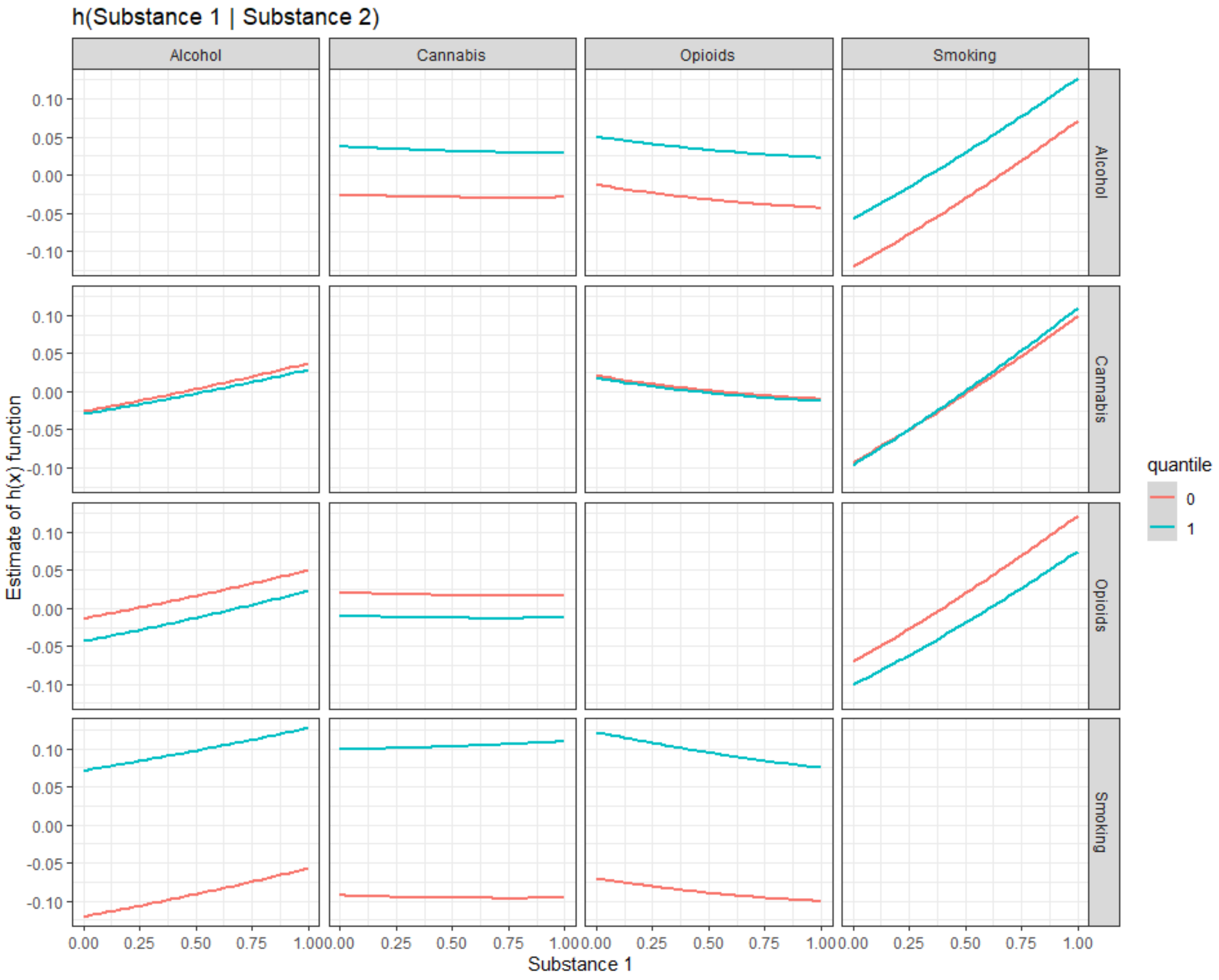

© 2022 Garrison-Desany HM et al. *JAMA Network Open*.

eFigure 5. Schoenfeld Residuals of Multiple Imputation Data (Data Set 1)

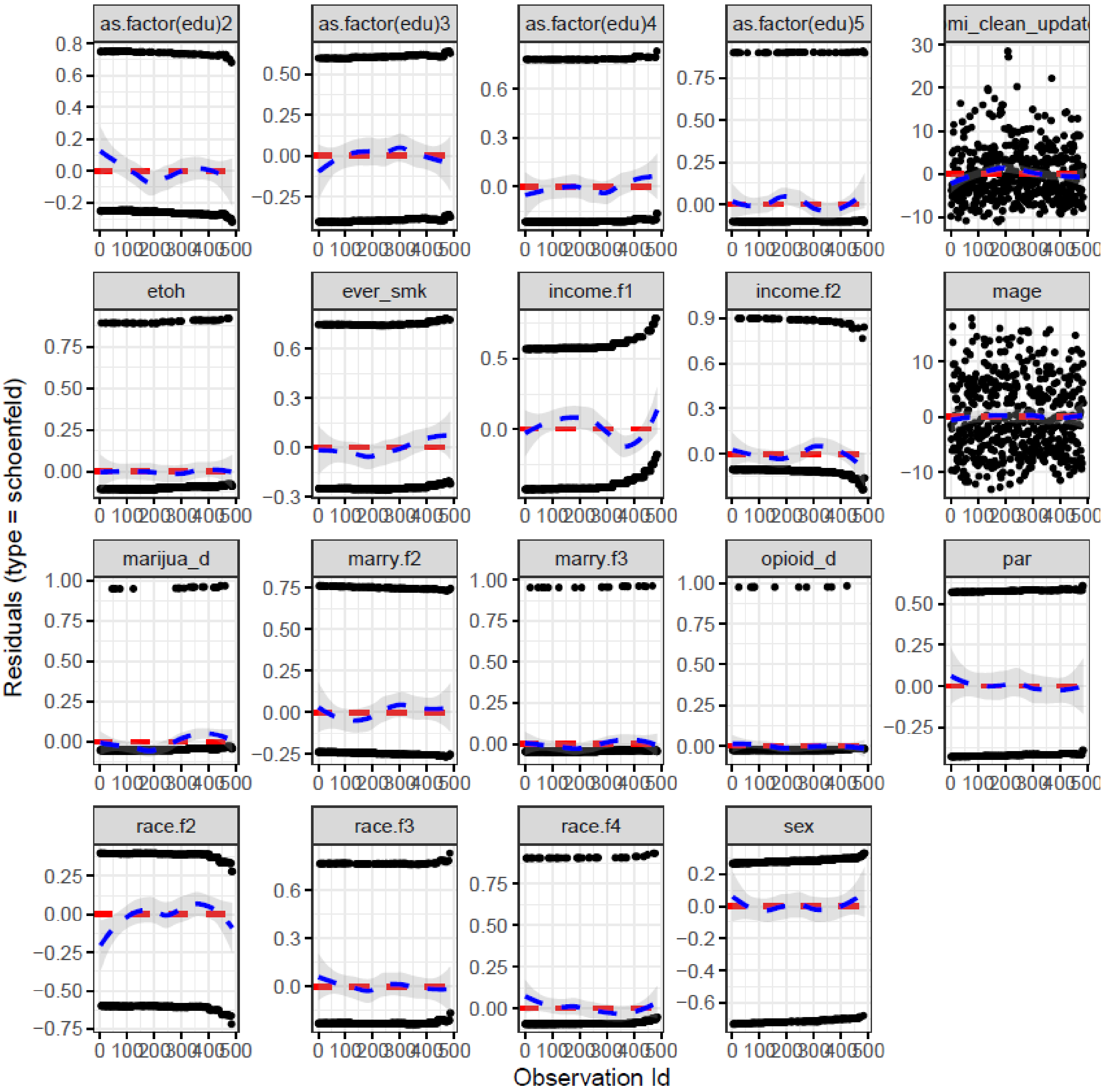

© 2022 Garrison-Desany HM et al. *JAMA Network Open*.

eFigure 6. Schoenfeld Residuals of Multiple Imputation Data (Data Set 2)

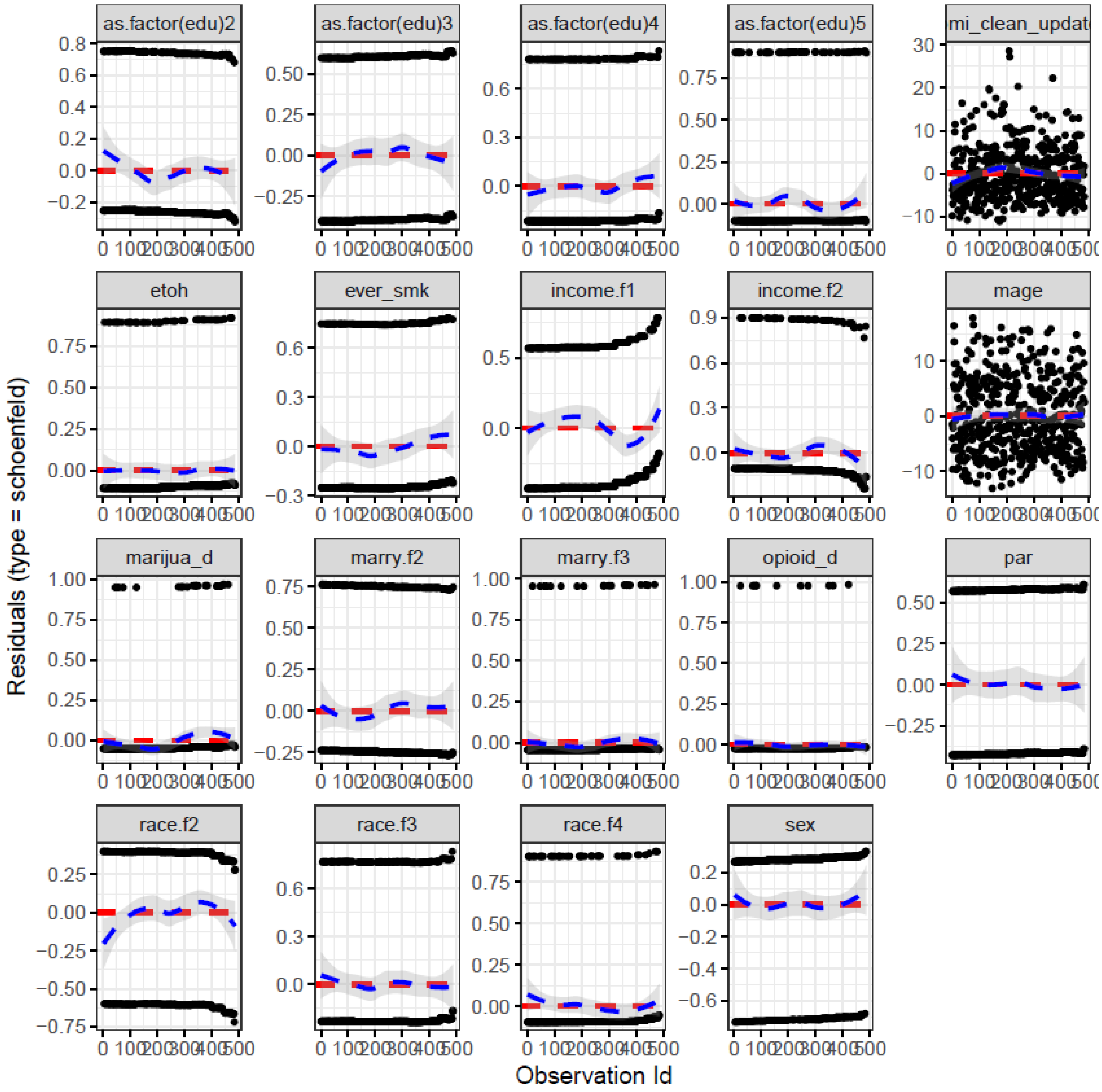

© 2022 Garrison-Desany HM et al. *JAMA Network Open*.

eFigure 7. Schoenfeld Residuals of Multiple Imputation Data (Data Set 3)

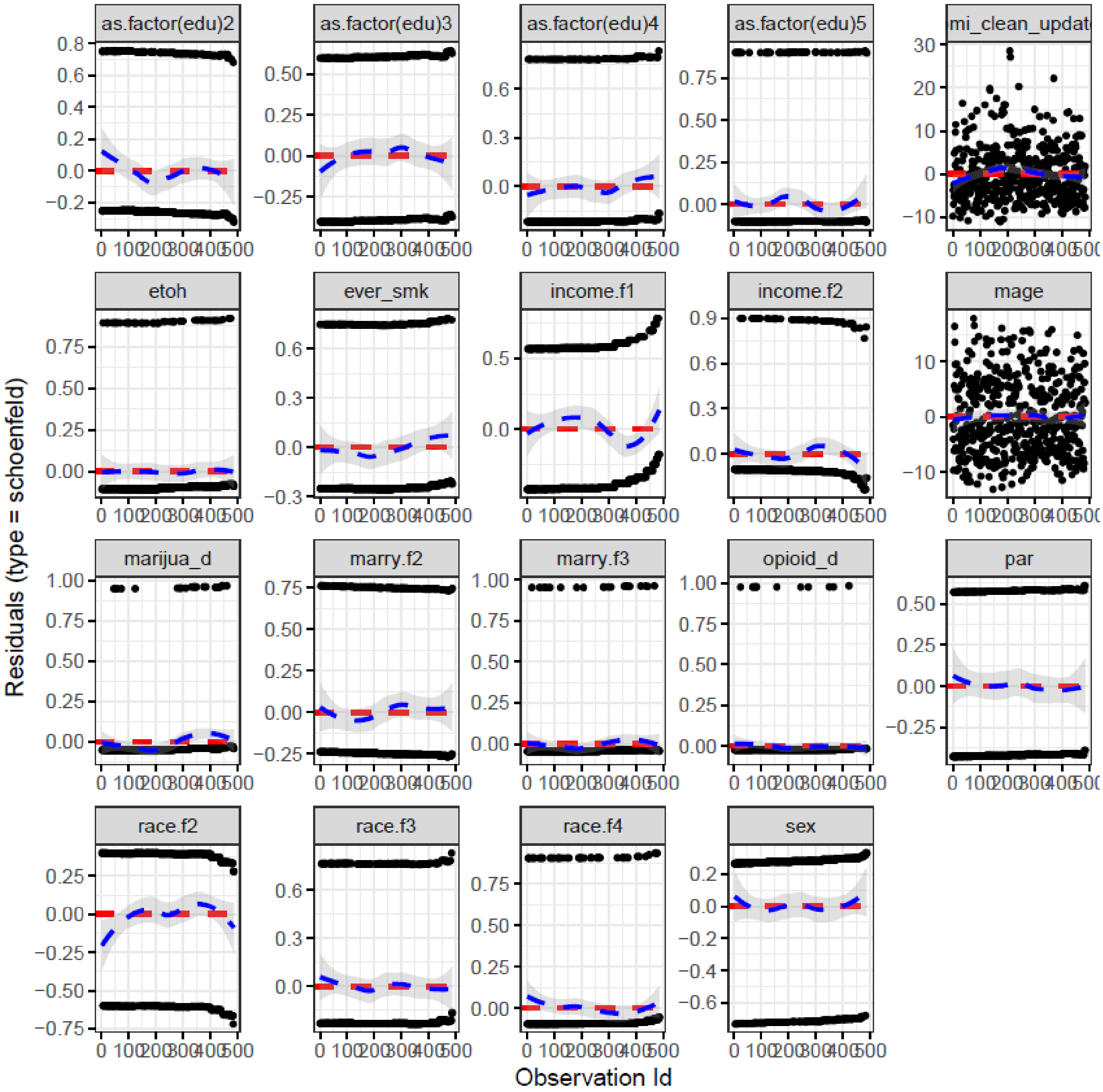

© 2022 Garrison-Desany HM et al. *JAMA Network Open*.

eFigure 8. Schoenfeld Residuals of Multiple Imputation Data (Data Set 4)

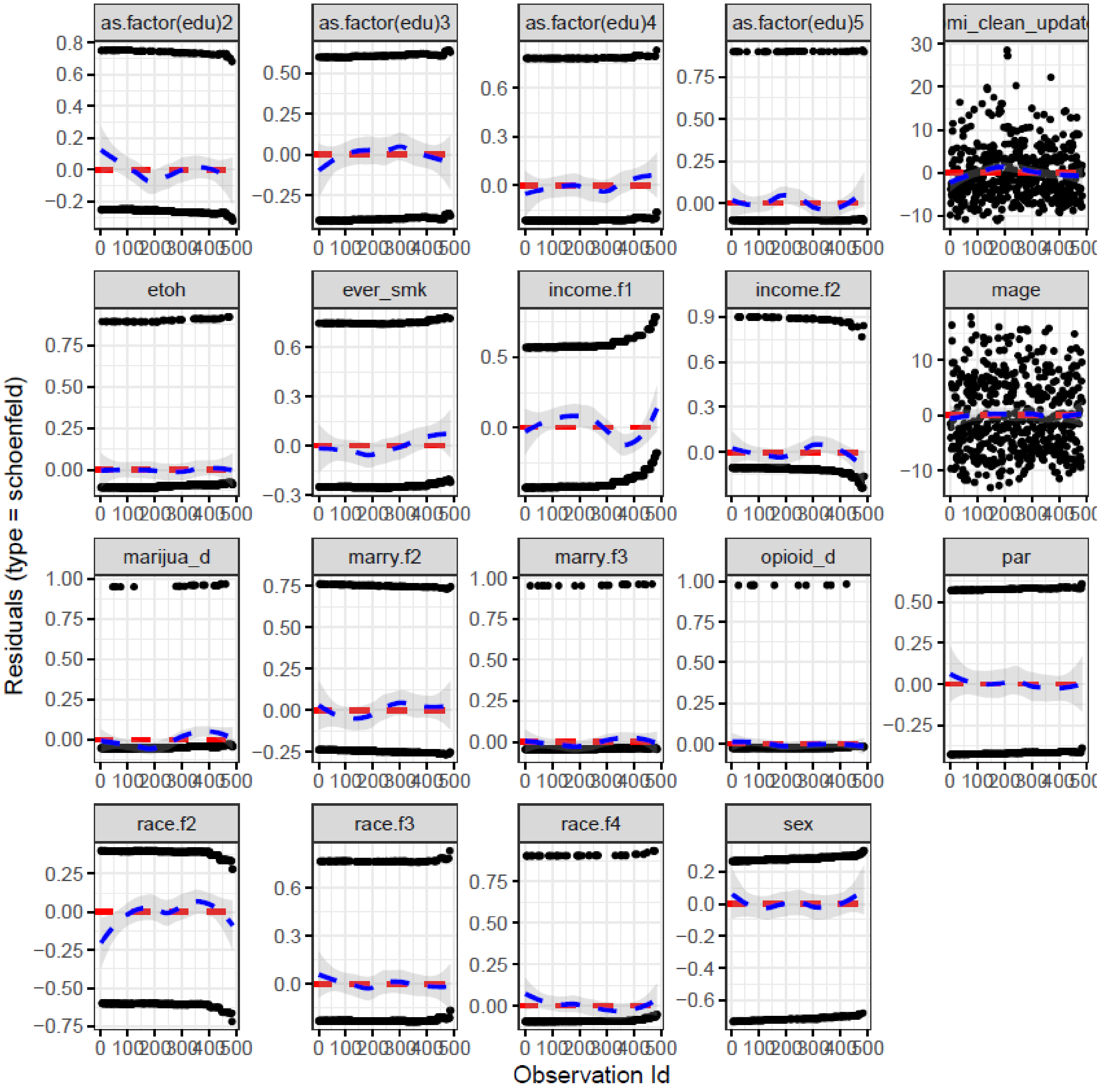

© 2022 Garrison-Desany HM et al. *JAMA Network Open*.

eFigure 9. Schoenfeld Residuals of Multiple Imputation Data (Data Set 5)

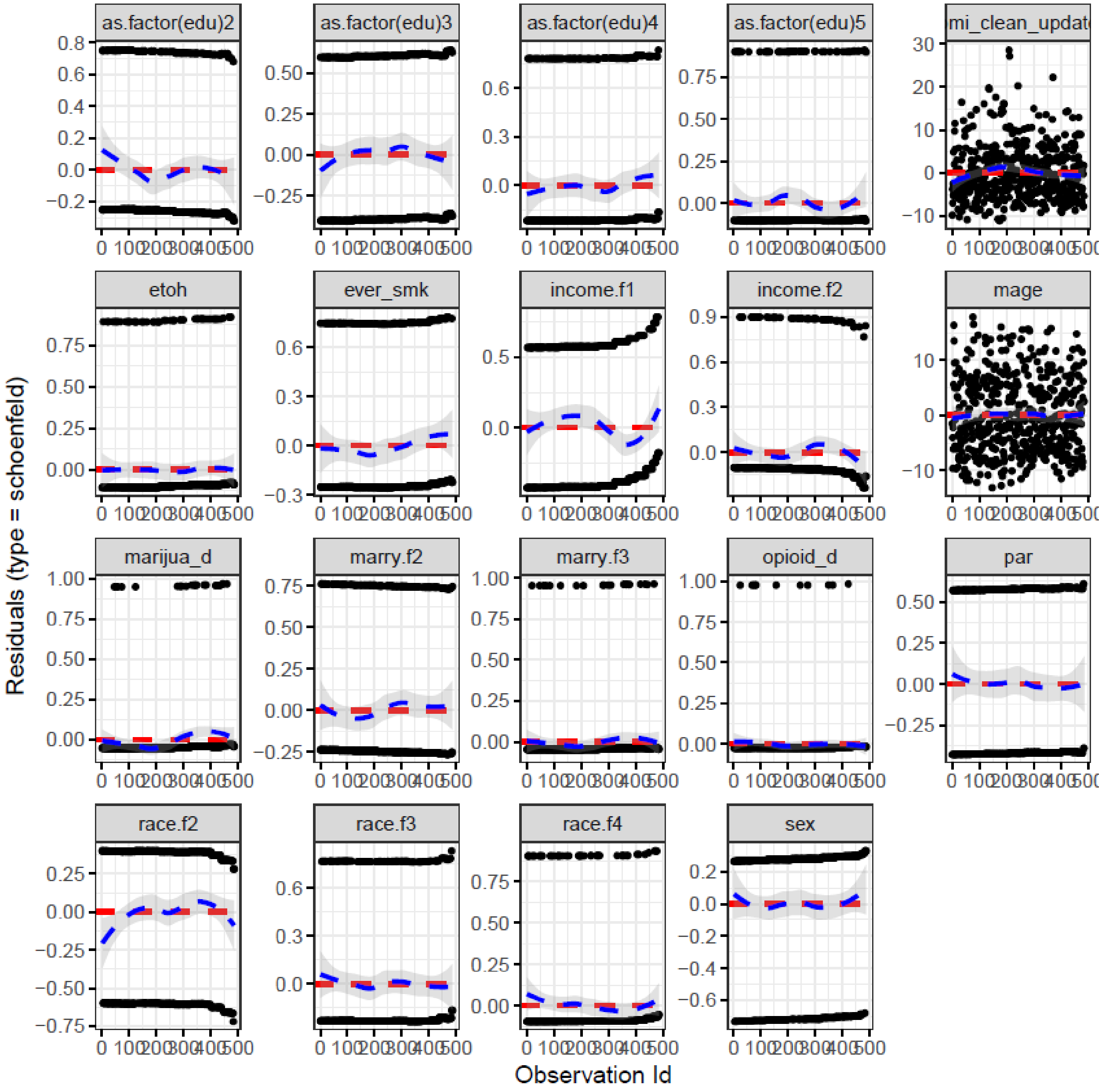

© 2022 Garrison-Desany HM et al. *JAMA Network Open*.

eFigure 10. Schoenfeld Residuals of Multiple Imputation Data (Data Set 6)

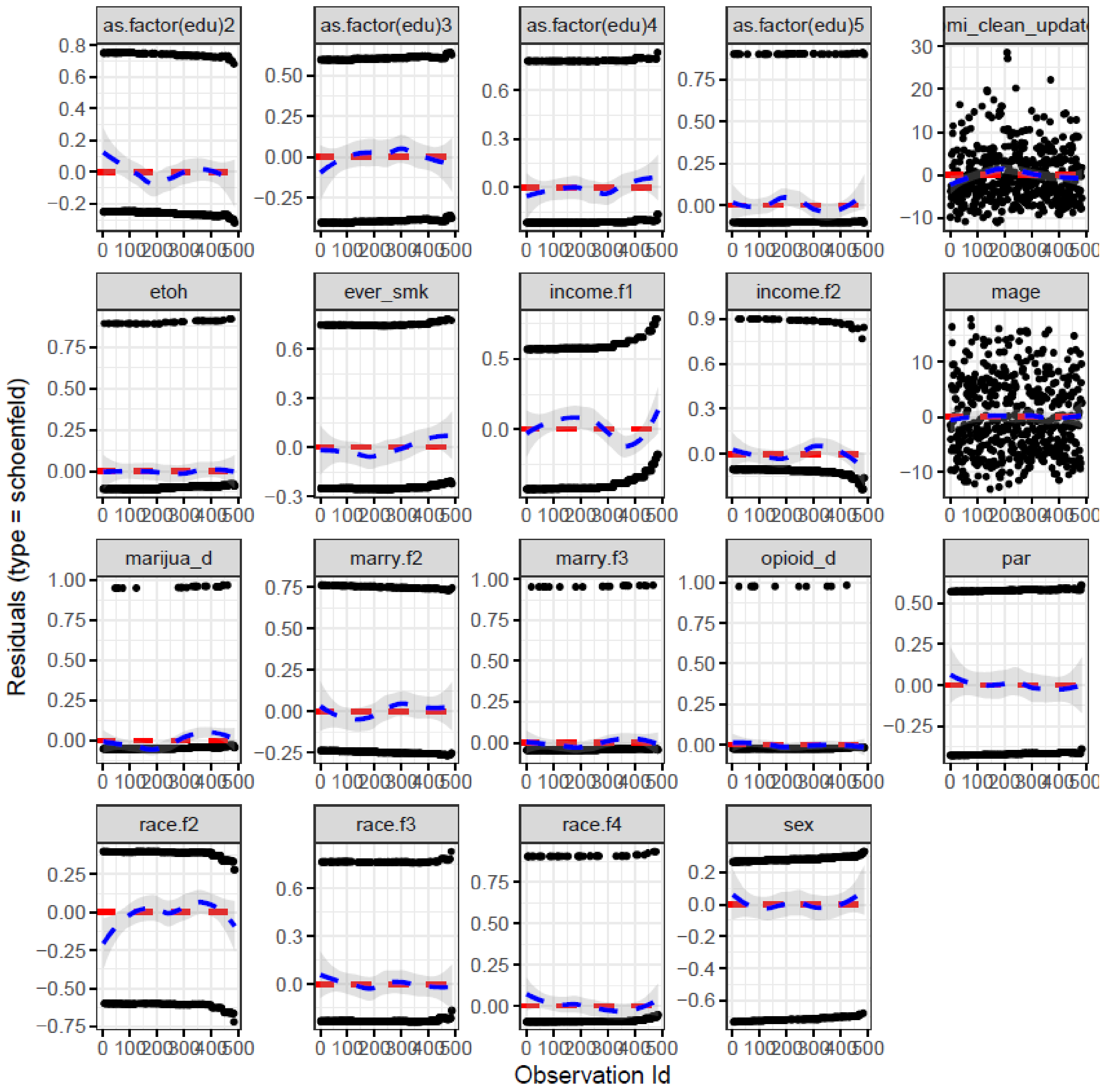

© 2022 Garrison-Desany HM et al. *JAMA Network Open*.

eFigure 11. Schoenfeld Residuals of Multiple Imputation Data (Data Set 7)

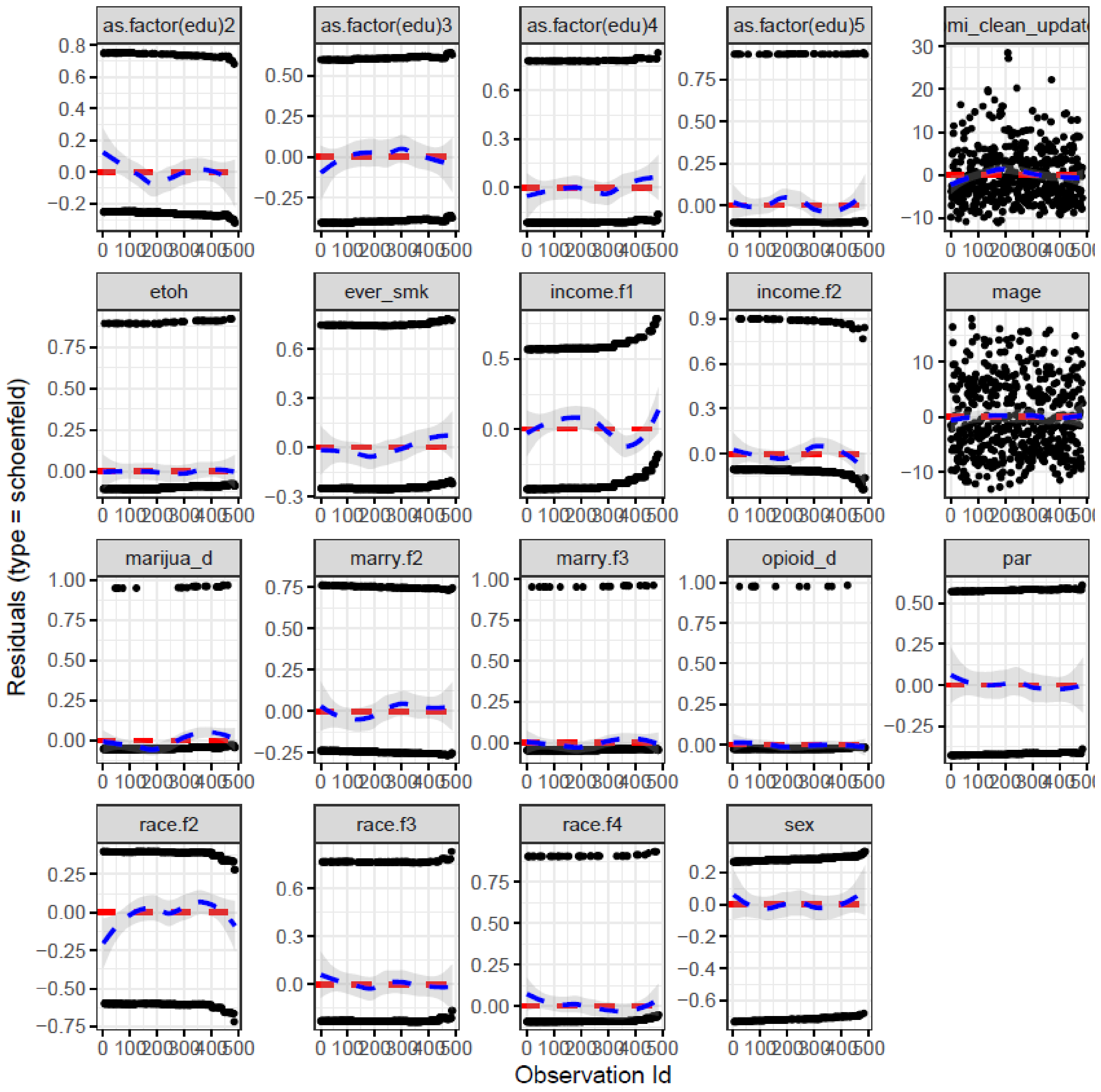

© 2022 Garrison-Desany HM et al. *JAMA Network Open*.

eFigure 12. Schoenfeld Residuals of Multiple Imputation Data (Data Set 8)

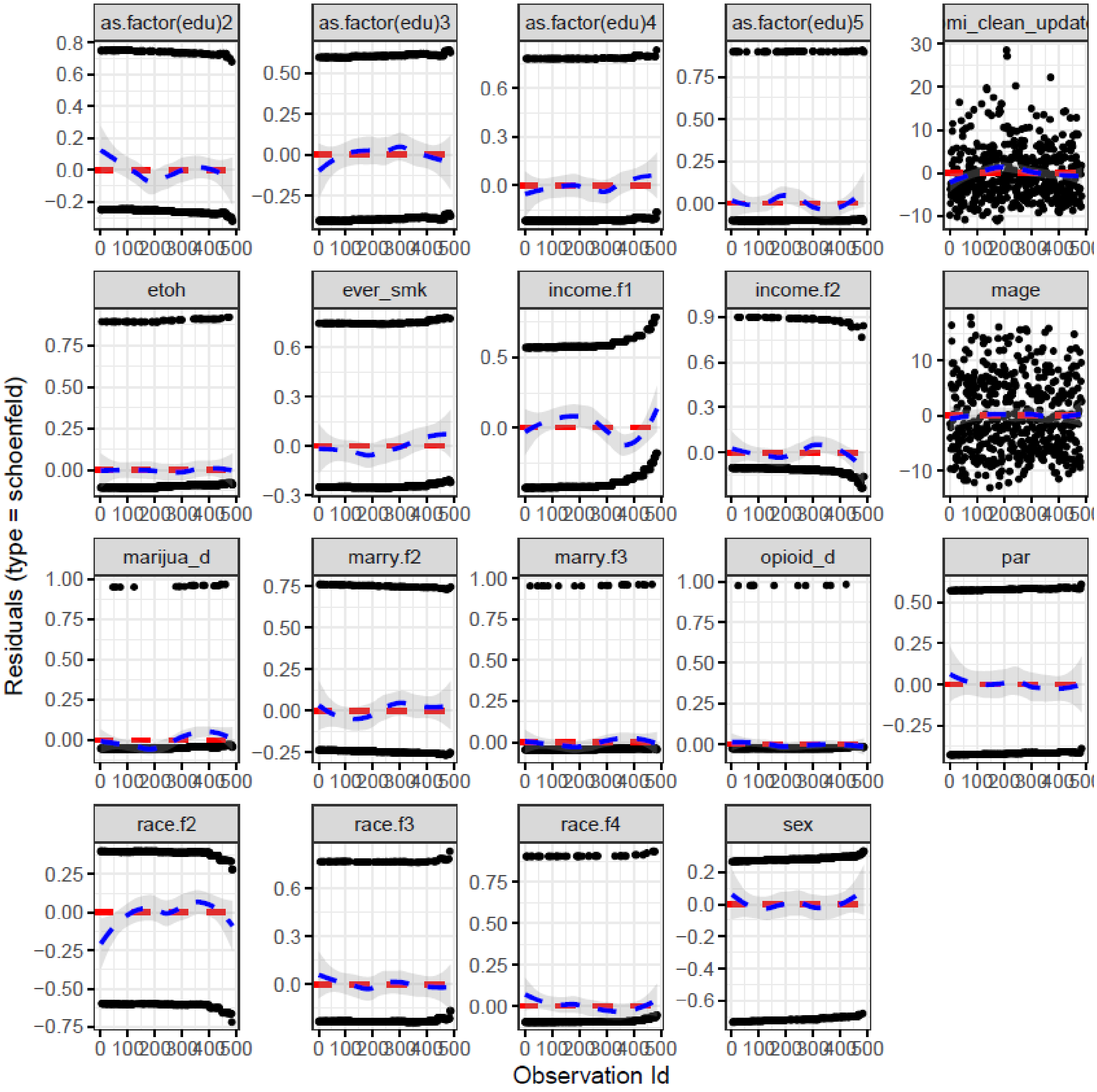

© 2022 Garrison-Desany HM et al. *JAMA Network Open*.

eFigure 13. Schoenfeld Residuals of Multiple Imputation Data (Data Set 9)

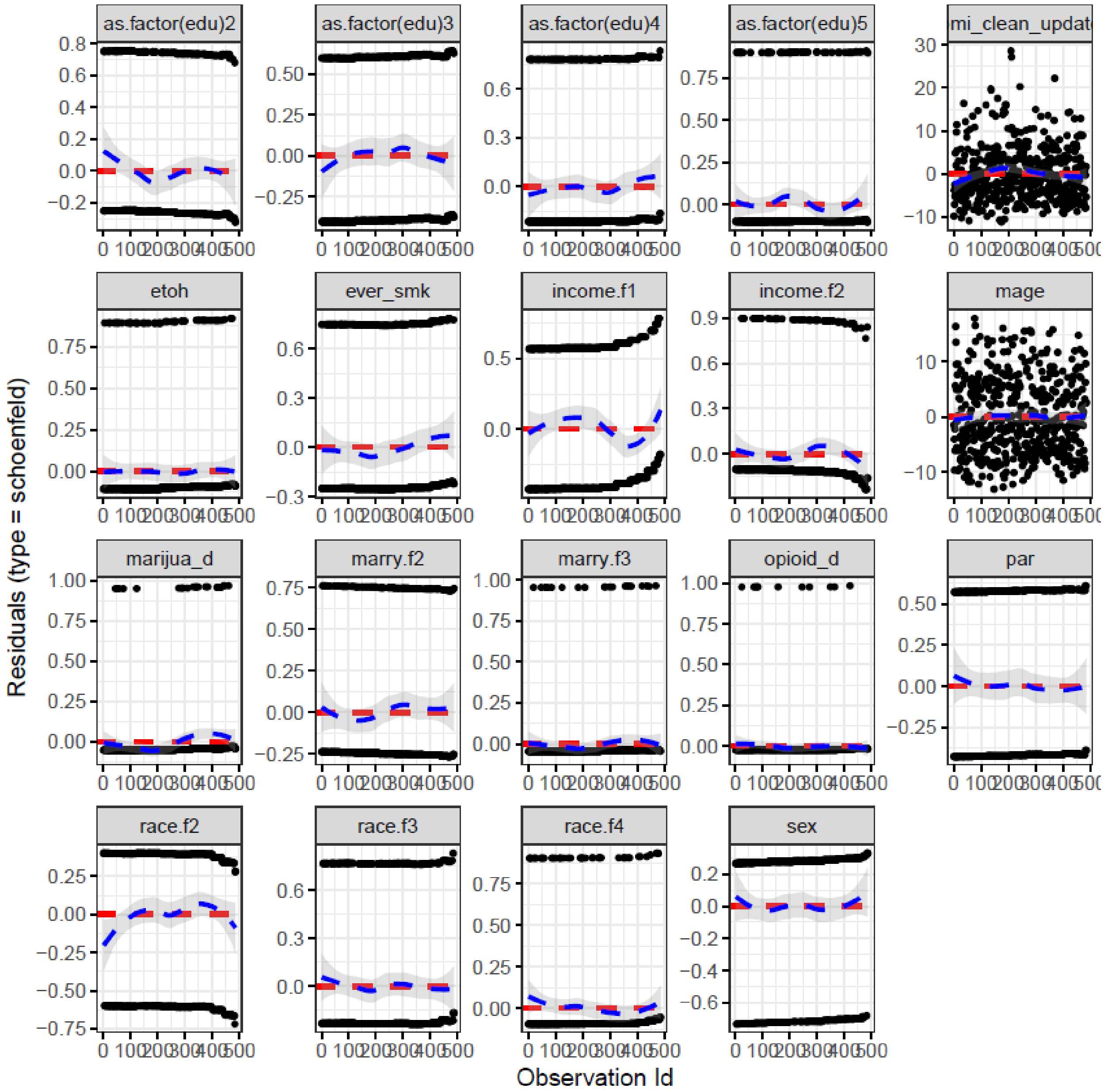

© 2022 Garrison-Desany HM et al. *JAMA Network Open*.

eFigure 14. Schoenfeld Residuals of Multiple Imputation Data (Data Set 10)

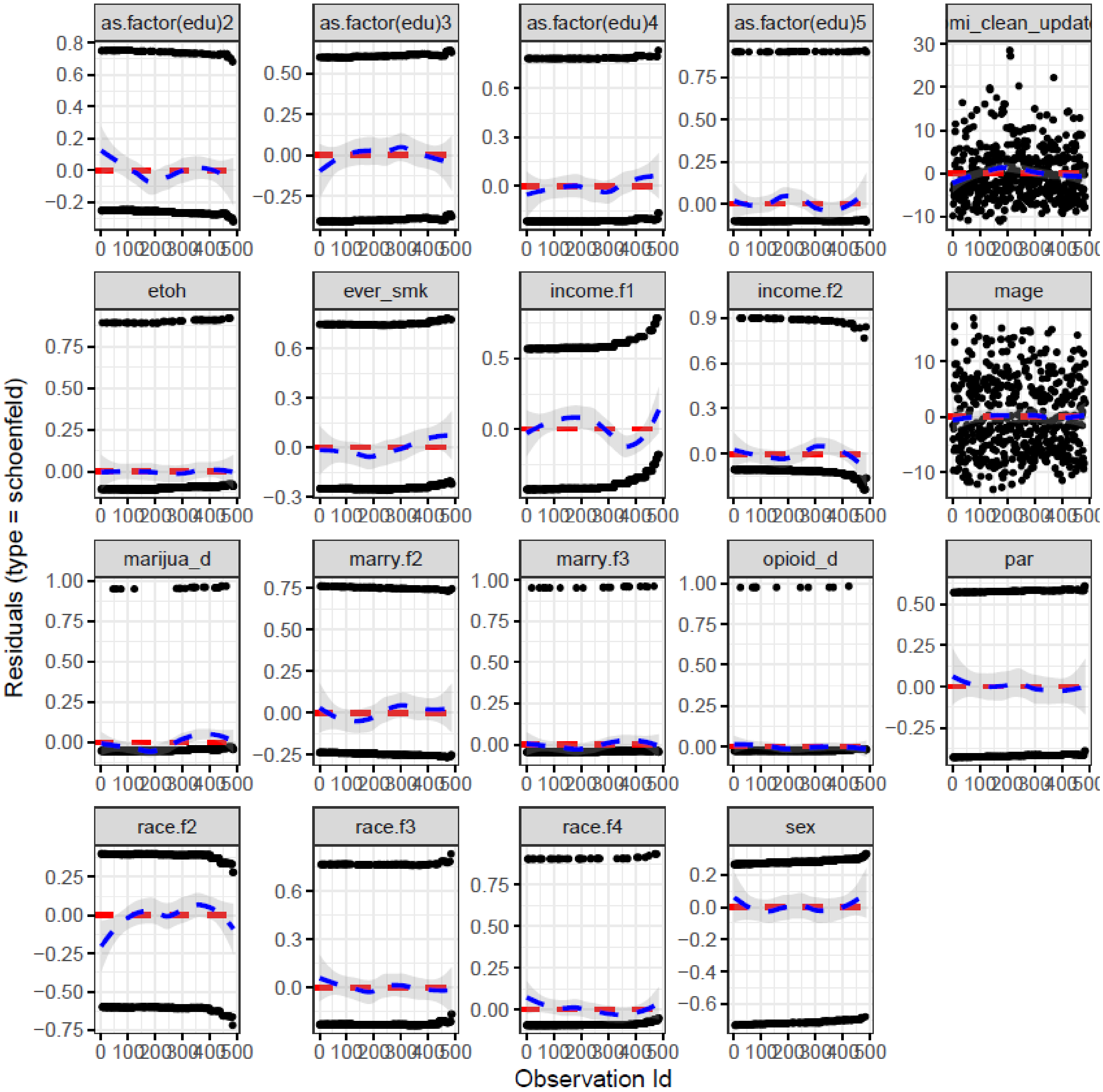

© 2022 Garrison-Desany HM et al. *JAMA Network Open*.
